# A Photoswitchable Chloride‐Binding [2]Rotaxane

**DOI:** 10.1002/chem.202500461

**Published:** 2025-04-08

**Authors:** Jorn de Jong, Sander J. Wezenberg

**Affiliations:** ^1^ Leiden Institute of Chemistry Leiden University Einsteinweg 55, 2333 CC Leiden The Netherlands

**Keywords:** anion binding, molecular switches, photochromism, rotaxanes, stiff‐stilbene

## Abstract

Control over the binding properties of anion receptors by external stimuli can be advantageous in various applications such as extraction and transport processes. Toward a light‐responsive anion receptor with high binding affinity and selectivity, a stiff‐stilbene photoswitch is incorporated into the macrocycle of a mechanically interlocked, chloride‐binding [2]rotaxane structure. UV‐Vis and ^1^H NMR studies show reversible transformation between *Z*/*E*‐isomers upon light irradiation, causing changes in motional dynamics and binding affinity. Photoswitching also takes place in the presence of chloride, as monitored by ^1^H NMR spectroscopy, which results in its concomitant uptake and release. Our results show the suitability of rotaxanes as light‐responsive ion receptors, which could serve as prototypes for supramolecular pumps.

## Graphical abstract

A [2]rotaxane receptor for chloride containing a stiff‐stilbene photoswitch in the macrocyclic ring can be switched between *Z*‐ and *E*‐isomers using 340/385 nm light. This brings about a change in motional dynamics as well as binding affinity. The presented approach is expected to facilitate photocontrolled extractions and transport with high chloride selectivity.

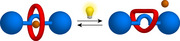

## 1 Introduction

Over the past decades, synthetic anion receptors^[^ ^1^ ^]^ have gained significant attention because of their potential to extract pollutants from wastewater,^[^ ^2^ ^]^ sense analytes,^[^ ^3^ ^]^ and mediate trans‐membrane transport.^[^ ^4^ ^]^ For these applications, control over the receptor's binding properties could be beneficial, for example, to strip the extracted substrate (and recycle the receptor) or to switch transport activity. Therefore, the development of stimuli‐responsive anion receptors is gathering major interest.^[^ ^5^ ^]^ In this regard, light has often been used as a stimulus to modulate anion binding affinity because of its high spatiotemporal precision and non‐invasive nature.^[^ ^6^ ^]^ Previously reported light‐switchable anion receptors have primarily been based on molecular tweezers,^[^ ^7^ ^]^ foldamers,^[^ ^8^ ^]^ and macrocycles.^[^ ^9^ ^]^ In our attempt to achieve stronger and more selective binding with such receptors, we recently developed a mechanically interlocked [2]catenane in which chloride uptake and release could be successfully controlled by light.^[^ ^10^ ^]^ It would be attractive to extend this concept towards rotaxanes, as they generally allow for more straightforward structural modification, including the installation of different stoppering groups, which has shown to be highly valuable in the development of molecular machines.^[^ ^11^ ^]^ While the group of Beer triggered anion binding to a [2]rotaxane by the addition of acid,^[^ ^12^ ^]^ to our best knowledge, the control of anion complexation by light in such structures has not yet been achieved.

**Scheme 1:**
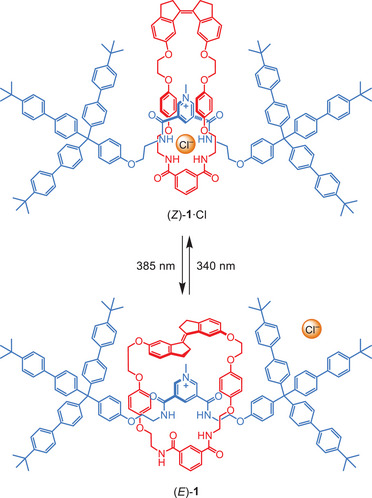
Schematic representation of light‐induced chloride binding and release by [2]rotaxane receptor **1**·PF_6_.

Taking inspiration from the seminal work by the group of Beer on anion‐templating of mechanically interlocked structures,^[^ ^13^, ^14^ ^]^ we previously incorporated a stiff‐stilbene photoswitch in an isophthalamide‐containing macrocycle, which allowed control of pseudorotaxane assembly with pyridinium halide axles.^[^ ^15^ ^]^ This photoswitch was shown beneficial to expand and contract macrocycles,^[^ ^16^ ^]^ as it has a high thermal stability and it undergoes a large change in geometry upon *E*/*Z* isomerization.^[^ ^17^ ^]^ For the *Z*‐isomer of our macrocycle, it was found that the halide ion could bind in between this macrocycle and the axle to stabilize the pseudorotaxane complex. With the *E*‐isomer, however, a stable threaded species could not be formed because of steric constraints. Where in this case the axle component was substituted with hexyl chains, we envisioned replacing them for bulky stoppers so that instead of assembly and disassembly, only the central anion would be bound and released upon photostimulation (Scheme 1).

Herein, we report the photoswitchable [2]rotaxane **1**·PF_6_ (see Scheme 1). Its *Z*‐isomer has been synthesized via an anion‐templated clipping approach using a bulky‐stoppered pyridinium chloride axle and the precursor to our stiff‐stilbene‐containing macrocycle. In this *Z*‐isomer, the mechanically interlocked components form a binding pocket with four amide hydrogen‐bond donors, which is shown to be suitable for accommodating chloride. The respective *E*‐isomer can be generated by irradiation with light, which alters the rotaxane's motional dynamics and reduces the chloride binding affinity. Importantly, the isomerization process is reversible and can be carried out in the presence of chloride, which facilitates its successful uptake and release. This work is a first example of a photoswitchable rotaxane‐based receptor and will stimulate the further development of responsive mechanically interlocked host–guest systems.

## 2 Results and Discussion

**Scheme 2:**
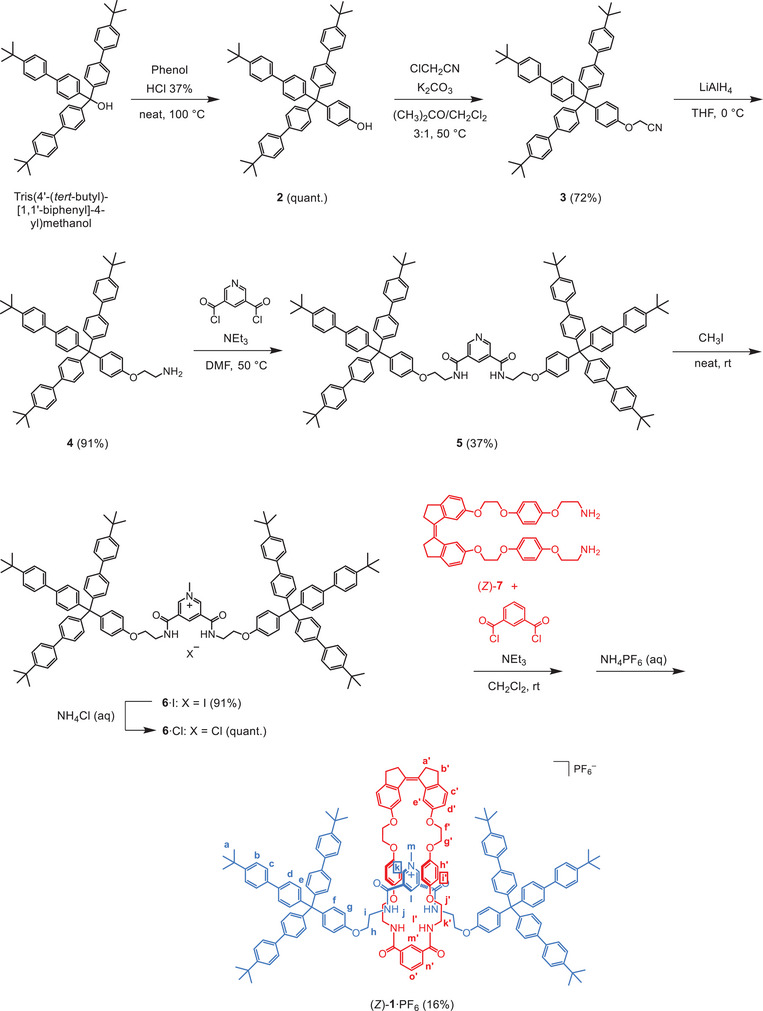
Synthesis of rotaxane (*Z*)‐**1**·PF_6_ via an anion‐templated clipping approach.

Rotaxane (*Z*)‐**1**·PF_6_ was prepared using an anion‐templated clipping approach (see Scheme 2), which was developed by the group of Beer and extensively used for the synthesis of anion‐binding mechanically interlocked molecules.^[^ ^13^, ^14^ ^]^ First, previously reported tris(4′‐(*tert*‐butyl)‐[1,1′‐biphenyl]‐4‐yl)methanol^[^ ^18^ ^]^ was reacted with phenol under acidic conditions to obtain compound **2**. Treatment with chloroacetonitrile in the presence of potassium carbonate as the base then gave nitrile‐substituted **3**. The nitrile group was reduced using lithium aluminum hydride to give amine derivative **4**, which was coupled with pyridine‐3,5‐dicarbonyl dichloride using triethylamine as the base to yield pyridine dicarboxamide **5**. Subsequent *N*‐methylation with iodomethane afforded pyridinium iodide axle **6**·I, after which the counteranion was exchanged for chloride by extensive washing with an aqueous ammonium chloride solution. Finally, the hence obtained axle **6**·Cl was used to template the cyclization between our previously reported stiff‐stilbene diamine (*Z*)‐**7** ^[^ ^15^ ^]^ and isophthaloyl dichloride. Next, the chloride anion was exchanged for the non‐coordinating hexafluorophosphate anion by washing with an aqueous ammonium hexafluorophosphate solution, giving the desired (*Z*)‐**1**·PF_6_ in a total yield of 16% over these last two steps.

The mechanically interlocked nature of (*Z*)‐**1**·PF_6_ was confirmed with 2D NMR spectroscopy and high resolution mass spectrometry (HRMS). The (^1^H,^1^H)‐ROESY spectrum of (*Z*)‐**1**·PF_6_ in DMSO‐*d*_6_ revealed through space interactions between protons of the macrocycle and of the axle (Figure S20 in the Supporting Information). For instance, cross‐peaks were found between the hydroquinone protons of the macrocycle (H_h’_ and H_i’_) and several stopper protons of the axle (H_d_, H_e_, and H_g_), indicating that the components were interlocked. The HRMS spectrum of (*Z*)‐**1**·PF_6_ featured a signal whose *m*/*z* ratio corresponded to the positively charged fragment containing the interlocked macrocycle and the axle without the hexafluorophosphate counteranion (*m*/*z*: 2450.2938, *m/z* calcd.: 2450.2874, see Figure S21 in the Supporting Information).

**Figure 1:**
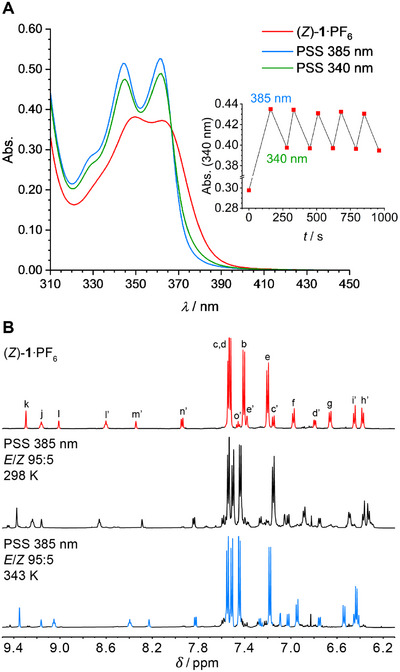
A) UV‐Vis spectral changes of (*Z*)‐**1**·PF_6_ upon consecutive irradiation with 385 nm and 340 nm light (*c* = 2.0 × 10^−5^ M in DMSO) and change in absorption at 340 nm during 385/340 nm irradiation cycles (inset). (B) ^1^H NMR spectra of a solution of (*Z*)‐**1**·PF_6_ (0.2 mM in DMSO‐*d*_6_, top), which was irradiated with 385 nm (middle) and subsequently heated to 343 K (bottom).

The photoswitching behavior of rotaxane (*Z*)‐**1**·PF_6_ was first studied by UV‐Vis spectroscopy in DMSO. Irradiation of a solution of this *Z*‐isomer with 385 nm light induced a shift of its initial absorption maxima (*λ* = 350 nm and 362 nm) toward shorter wavelengths (*λ* = 344 nm and 362 nm; see Figure 1A and Figures S22 and S23 in the Supporting Information). Besides, the overall absorption increased, which, together with the hypsochromic shift of the absorption maxima, is indicative of *Z*→*E* isomerization.^[^ ^16^, ^17^ ^]^ Subsequent irradiation of the same sample with 340 nm light resulted in a bathochromic shift and an overall decrease of the absorption, illustrative of isomerization back to the *Z*‐isomer. With both wavelengths, irradiation was continued until the photostationary states (PSS) were reached, and, meanwhile, an isosbestic point was observed at *λ* = 366 nm, which corroborated the unimolecular nature of the photoisomerization process. The photoswitching cycle using 385 and 340 nm light could be repeated multiple times without significant signs of photofatigue (see Figure 1A, inset).

Next, the photoisomerization process was monitored by ^1^H NMR spectroscopy in DMSO‐*d*_6_. After a solution of (*Z*)‐**1**·PF_6_ was irradiated with 385 nm light, a new set of signals emerged, which could be assigned to the *E*‐isomer (see Figure 1B and Figures S24–S26 in the Supporting Information). For example, the signals of axle protons H_k_, H_j_, and H_l_, which were originally located at *δ* = 9.29, 9.16, and 9.01 ppm, now appeared at *δ* = 9.37, 9.24, and 9.16 ppm, respectively. Furthermore, the macrocycle isophthalamide proton signals H_l’_ and H_m’_ shifted from *δ* = 8.60 and 8.34 ppm to *δ* = 8.66 and 8.29 ppm. Interestingly, these new signals of the *E*‐isomer were broader than the original ones belonging to the *Z*‐isomer, and furthermore, minor secondary signals seemed to be present in the spectrum. When a ^1^H NMR spectrum of this PSS_385_ mixture was taken at a higher temperature (343 K), the proton signals of (*E*)‐**1**·PF_6_ sharpened and the secondary signals largely disappeared (see Figure 1B and Figures S27 and S28 in the Supporting Information). Most likely, the signal broadening at room temperature originates from slow interchange between different possible conformations of rotaxane (*E*)‐**1**·PF_6_. Hence, photoisomerization has a profound effect on the relative motional dynamics of the macrocycle and the axle. That is, shuttling and circumrotation of the macrocycle relative to the axle appear to become slower upon *Z*→*E* isomerization. Lastly, the PSS_385_ mixture was irradiated with 340 nm light, showing partial isomerization back to the Z‐isomer (Figures S24–S26 in the Supporting Information). Comparison of the integrals of the ^1^H NMR signals of (*E*)‐**1**·PF_6_ and (*Z*)‐**1**·PF_6_ allowed calculation of the PSS_385_ and PSS_340_ (*E*/*Z*)‐ratios, which gave 95:5 and 65:35, respectively. These ratios are similar to those found for other cyclized stiff‐stilbene photoswitches reported previously.^[^ ^9^, ^16^ ^]^

The chloride binding affinity of both the *Z*‐ and *E*‐isomer of **1**·PF_6_ was quantified by ^1^H NMR titrations in DMSO‐*d*_6_/0.5% H_2_O. When a solution of tetrabutylammonium chloride (NBu_4_Cl) was added to (*Z*)‐**1**·PF_6_, substantial spectral changes were observed, in particular for the signals of the protons directly involved in anion binding (i.e., the NH and aromatic CH protons of the axle's pyridinium group and the macrocycle's isophthalamide moiety; H_j_, H_l_, H_l’_, and H_m’_). After the addition of approximately 20 equivalents of NBu_4_Cl (at which point saturation was nearly reached), proton signals H_l_, H_l’_, and H_m’_ shifted downfield from *δ* = 9.01, 8.60, and 8.34 ppm to *δ* = 9.19, 8.75, and 8.84 ppm, respectively (Figures S29 and S30 in the Supporting Information). At the same time, amide proton signal H_j_ shifted upfield, from *δ* = 9.16 ppm to *δ* = 8.98 ppm. The shifting of these proton signals was fitted to a 1:1 binding equilibrium with HypNMR software,^[^ ^19^ ^]^ giving a stability constant (*K*_a,_ *_Z_*) of 4.9 × 10^2^ M^−1^. This value is similar to what we have determined previously for chloride binding to our photoswitchable [2]catenane receptor [(*K*_a,_ *_Z_*) of 6.0 × 10^2^ M^−1^].^[^ ^10^ ^]^

When a similar ^1^H NMR titration was carried out with (*E*)‐**1**·PF_6_, the chemical shift changes were found to be significantly smaller, and 20 equivalents of NBu_4_Cl were not sufficient to reach binding saturation (Figures S31 and S32 in the Supporting Information). Fitting of the data for this isomer to a 1:1 binding model using HypNMR resulted in a stability constant (*K*_a,_ *_E_*) of 1.5 × 10^2^ M^−1^, which is more than three times lower than what was determined for the *Z*‐isomer. Thus, *Z*→*E* photoisomerization of **1**·PF_6_ leads to a modest decrease in chloride binding affinity. This decrease is most likely due to increased steric congestion at the anion binding site. That is, the geometry change upon *Z*→*E* isomerization of the photoswitchable macrocycle causes the distance between its stiff‐stilbene and isophthalamide moieties to reduce. Therefore, a suitable chloride binding pocket cannot be formed together with the pyridinium bis‐amide motif on the axle, similar to what we observed recently for our catenated system, which showed a 5‐fold decrease in chloride affinity in DMSO.^[^ ^10^ ^]^

**Figure 2:**
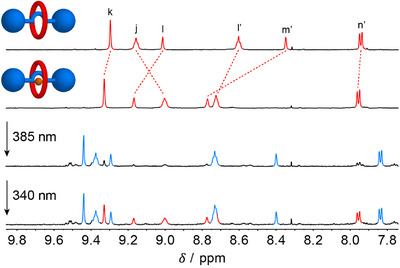
^1^H NMR spectral changes upon addition of 20 equivalents of NBu_4_Cl to (*Z*)‐**1**·PF_6_ (0.2 mM in DMSO‐*d*_6_), followed by irradiation with 385 nm and 340 nm light.

Finally, photoisomerization in the presence of chloride and hence, its concomitant binding and release was followed by ^1^H NMR spectroscopy (Figure 2 and Figure S33 in the Supporting Information). First, 20 equivalents of NBu_4_Cl were added to a solution of (*Z*)‐**1**·PF_6_ in DMSO‐*d*_6_, upon which 72% of rotaxane was associated with chloride, as was calculated on the basis of the chemical shift of protons located in the binding site (H_j_, H_l_, H_l’_, and H_m’_), and is in agreement with the determined association constant (vide supra). Then, the mixture was irradiated with 385 nm light, which gave an (*E*/*Z*)‐ratio of 85:15 at the PSS. Now, only 59% of the *E*‐isomer was found to be associated with chloride due to its lower binding affinity. Part of the originally complexed chloride was thus released in solution. Switching back to the *Z*‐isomer with 340 nm light led to partial reuptake of chloride from the solution, and now an (*E*/*Z*)‐ratio of 60:40 was determined at the PSS. Interestingly, these PSS ratios in the presence of chloride are slightly more enriched in the Z‐isomer compared to the uncomplexed rotaxane (i.e., PSS_385_ and PSS_340_ (*E*/*Z*)‐ratios of **1**·PF_6_ were 95:5 and 65:35, respectively [vide supra]). Whether this difference is caused by an electronic and/or a nanomechanical effect is still unclear at this moment.

## 3 Conclusions

In summary, we have synthesized a mechanically interlocked [2]rotaxane receptor containing a stiff‐stilbene photoswitch. Reversible isomerization between its *Z*‐ and *E*‐isomer induced by 340/385 nm light was shown to affect motional dynamics as well as chloride binding affinity. Where the *Z*‐isomer showed strong chloride binding in the highly competitive DMSO/0.5% H_2_O solvent mixture, the *E*‐isomer had a 3‐fold lower binding affinity. Photoisomerization also took place in the presence of chloride, which slightly changed the PSS ratios in favor of the *Z*‐isomer and led to its concomitant uptake and release on demand. We expect this work to be a stepping stone in the development of light‐responsive rotaxane‐based receptors. Further fine‐tuning will be needed to improve the affinity difference between photoaddressable states and facilitate, for example, photocontrolled anion extraction from aqueous solutions. In addition, the use of different (pseudo)stoppering groups could enable the creation of anion‐templated supramolecular pumps,^[^ ^11^ ^]^ which we will demonstrate in the near future.

## 4 Experimental Section

### 4.1 Rotaxane (*Z*)‐1·PF_6_

Diamino‐functionalized stiff‐stilbene (*Z*)‐**7** ^[^ ^15^ ^]^ (20 mg, 32 µmol) and pyridinium chloride axle **6**·Cl (57 mg, 33 µmol) were dissolved in dry CH_2_Cl_2_ (7.3 mL) in an oven‐dried round‐bottom flask under an argon atmosphere. After stirring the solution for 10 min at rt, Et_3_N (11 µL, 79 µmol, 2.5 eq.) was added, which was immediately followed by the dropwise addition of a 45 mM solution of isophthaloyl chloride in dry CH_2_Cl_2_ (0.73 mL, 33 µmol). The reaction mixture was stirred for 1.5 h at rt and then diluted with CH_2_Cl_2_ to reach a total volume of 20 mL. The organic solution was washed with 1 M HCl aq. (2 × 10 mL) and water (2 × 10 mL), dried over anhydrous Na_2_SO_4_ and concentrated. Purification by column chromatography (SiO_2_; CH_2_Cl_2_/acetone 85:15 to 75:25) gave rotaxane (*Z*)‐**1**·Cl as a light yellow solid, which contained some minor impurities that were not removed prior to the anion exchange step. The material was redissolved in CHCl_3_ (5 mL), and 1 M NH_4_PF_6_ aq. (5 mL) was added. The biphasic mixture was stirred vigorously for 1 min at rt. The organic layer was separated, and this anion‐exchange step using the NH_4_PF_6_ solution was repeated two more times, after which the organic phase was washed with water (2 × 5 mL) and concentrated. The product was purified by precipitation from CH_2_Cl_2_/CH_3_CN to obtain rotaxane (*Z*)‐**1**·PF_6_ as a white solid (13 mg, 16%); *R*_f_ = 0.54 (SiO_2_; CH_2_Cl_2_/acetone 80:20); ^1^H NMR (600 MHz, DMSO‐*d*_6_; assignments are based on COSY and ROESY NMR spectra) *δ* = 9.30 (s, 2H; H_k_), 9.16 (t, *J* = 5.0 Hz, 2H; H_j_), 9.01 (s, 1H; H_l_), 8.60 (t, *J* = 5.2 Hz, 2H; H_l’_), 8.34 (s, 1H; H_m’_), 7.94 (dd, *J* = 7.8, 1.4 Hz, 2H; H_n’_), 7.53 (m, 24H; H_c_, H_d_), 7.46 (t, *J* = 7.8 Hz, 1H; H_o’_), 7.41 (d, *J* = 8.5 Hz, 12H; H_b_), 7.38 (d, *J* = 1.9 Hz, 2H; H_e’_), 7.20 (d, *J* = 8.4 Hz, 12H; H_e_), 7.15 (d, *J* = 8.3 Hz, 2H; H_c’_), 6.98 (d, *J* = 8.8 Hz, 4H; H_f_), 6.79 (dd, *J* = 8.3, 1.9 Hz, 2H; H_d’_), 6.66 (d, *J* = 8.8 Hz, 4H; H_g_), 6.45 (d, *J* = 9.0 Hz, 4H; H_i’_), 6.38 (d, *J* = 9.0 Hz, 4H; H_h’_), 4.36 (s, 3H; H_m_), 3.99–3.94 (m, 4H; H_f’_), 3.94–3.86 (m, 8H; H_h_, H_j’_), 3.84–3.78 (m, 4H; H_g’_), 3.61–3.53 (m, 8H; H_i_, H_k’_), 2.82–2.76 (m, 4H; H_b’_), 2.71–2.63 (m, 4H; H_a’_), 1.27 (s, 54H; H_a_) ppm; ^13^C NMR (214 MHz, DMSO‐*d*_6_; due to low quantity and solubility no ^13^C NMR could be recorded and chemical shifts were derived from (^1^H,^13^C)‐HSQC and (^1^H,^13^C)‐HMBC spectra) *δ* = 166.2, 161.9, 156.9, 156.0, 152.4, 151.9, 149.9, 146.1, 145.5, 141.0, 140.8, 140.5, 138.3, 137.5, 136.6, 135.2, 134.4, 131.3, 130.8, 129.8, 129.7, 128.2, 126.4, 126.2, 125.8, 125.7, 125.6, 114.8, 114.7, 113.3, 113.2, 110.3, 67.0, 66.5, 66.3, 65.2, 63.1, 48.1, 39.1 (2), 34.8, 34.2, 30.7, 28.9 ppm; ^19^F NMR (376 MHz, DMSO‐*d*_6_) *δ* = −70.1 (d, *J* = 711 Hz) ppm; ^31^P{^1^H} NMR (162 MHz, DMSO‐*d*_6_) *δ* = −144.2 (sept, *J* = 711 Hz) ppm; IR (ATR) *ν* = 3675 (br. very w), 3311 (br. w), 3085 (very w), 2959 (s), 2926 (s), 2855 (m), 1662 (m), 1606 (w), 1543 (m), 1508 (s), 1500 (m, sh), 1477 (m), 1463 (m), 1394 (m), 1364 (w), 1225 (s), 1186 (m), 1113 (m), 1066 (s), 1004 (m), 847 (s), 817 (s), 765 (very w), 733 (w), 669 (very w), 623 cm^−1^ (very w); HRMS (ESI) *m*/*z*: 2450.2938 ([M]^+^, calcd for C_168_H_170_N_5_O_12_^+^: 2450.2874).

## Supporting Information

General experimental procedures, procedures for the synthesis of compounds 2–6, compound characterization data (Figures S1–S19), UV‐Vis and ^1^H NMR irradiation experiments, VT‐NMR and ^1^H NMR titration experiments are provided in the Supporting Information. The authors have cited an additional reference within the Supporting Information.^[^ ^20^ ^]^

## Conflict of Interests

The authors declare no conflicts of interest.

## Supporting information



Supporting Information

## Data Availability

The data that support the findings of this study are available in the supporting information of this article.
