# Supplementary material for: A Photoswitchable Chloride‐Binding [2]Rotaxane
Source: Chemistry. 2025 Apr 8;31(25):e202500461. doi: 10.1002/chem.202500461 (PMC12057604; doi:10.1002/chem.202500461)
Supplement: Supplementary file 1 — Supporting Information [file CHEM-31-e202500461-s001.pdf]

Supporting Information

A Photoswitchable Chloride-Binding [2]Rotaxane

Jorn de Jong,[a] and Sander J. Wezenberg[a]*

[a] *Leiden Institute of Chemistry, Leiden University,*

Einsteinweg 55, 2333 CC Leiden, The Netherlands

Email: s.j.wezenberg@lic.leidenuniv.nl

Table of Contents

Experimental section S1

NMR spectra of new compounds S8

HRMS spectrum of title compound S19

UV-Vis irradiation experiments S20

^{1}H NMR irradiation experiments S21

Variable-temperature ^{1}H NMR experiments S23

^{1}H NMR titration experiments S24

^{1}H NMR irradiation in presence of chloride S27

References S28

Experimental section

General methods and materials

CH_2Cl_2 was dried using a Pure Solve 400 solvent purification system from Innovative Technology. Dry Et_3N, DMF and DMSO were purchased from Sigma Aldrich. DMSO-d_6 and $CDCl_3$ were purchased from Eurisotop. Where needed, solvents were degassed by purging with N_2 for 30 min unless noted otherwise. Tris(4'-(*tert*-butyl)-[1,1'-biphenyl]-4-yl)methanol[1], pyridine-3,5-dicarbonyl dichloride[2] and diamine-functionalized stiff-stilbene (*Z*)-**7**[3] were prepared according to procedures reported in the literature. All other chemicals were commercial products and were used without further purification. Column chromatography was performed using silica gel (SiO_2) purchased from Screening Devices BV (pore diameter 55-70 Å, surface area 500 m^2g^{-1}) and thin-layer chromatography (TLC) was carried out on aluminum sheets coated with silica 60 F254 obtained from Merck. Compounds were visualized with UV light (254 nm). Melting points were determined with a Büchi M-560 apparatus. ^1H, ^{13}C, ^{19}F and ^{31}P NMR spectra were recorded on Bruker AV 400WB, Bruker AV 600 and Bruker AV 850 instruments at the indicated temperature. Chemical shifts (δ) are denoted in parts per million (ppm) relative to residual protiated solvent (DMSO-d_6: for ^1H detection, $\delta = 2.50$ ppm; for ^{13}C detection, $\delta = 39.52$ ppm; $\delta = 77.16$ ppm; $CDCl_3$: for ^1H detection, $\delta = 7.26$ ppm; for ^{13}C detection, $\delta = 77.16$ ppm). The splitting pattern of peaks is designated as follows: s (singlet), d (doublet), t (triplet), q (quartet), dd (doublet of doublets), m (multiplet), br. (broad). IR spectra were recorded on a Perkin Elmer Spectrum Two FT-IR spectrometer. The intensity of bands (v = cm^{-1}) is assigned as follows: s (strong), m (medium), w (weak), very w (very weak), br (broad), and sh (shoulder). High-resolution mass spectrometry (ESI-MS) was performed on a Thermo Scientific Q Exactive HF spectrometer with ESI ionization. UV-Vis spectra were recorded on an Agilent Cary 8454 spectrometer in a 1 cm quartz cuvette. Irradiation of UV-Vis and NMR samples was carried out using Thorlabs model M340F3 (1.06 mW), M340L4 (60 mW) and M385F1 (10.7 mW) LEDs, positioned at a distance of 1 cm to the sample.

4-(tris(4'-(*tert*-butyl)-[1,1'-biphenyl]-4-yl)methyl)phenol (2):

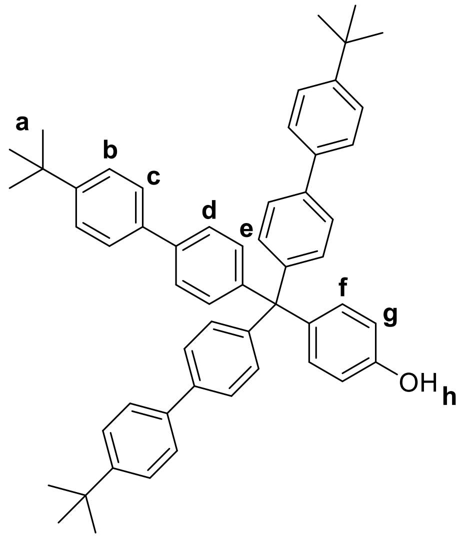

Tris(4'-(*tert*-butyl)-[1,1'-biphenyl]-4-yl)methanol (0.60 g, 0.91 mmol) was dissolved in an excess of phenol (10 g) at 70 °C. Then, 37% HCl aq. (0.1 mL) was added and the temperature was increased to 100 °C. After stirring for 17 h, the mixture was allowed to cool to rt and diluted with toluene (50 mL). The organic layer was washed with 1 M NaOH aq. (4 × 50 mL), 1 M HCl aq. (2 × 50 mL), and water (2 × 50 mL), dried over anhydrous Na_2SO_4 and concentrated to give **2** as an off-white solid (0.67 g, 100%) which was used without further purification; R_f = 0.20 (SiO_2; CH_2Cl_2/pentane 1:1); m.p. 345 – 346 °C; ^{1}H NMR (400 MHz, $CDCl_3$; assignments are based on COSY and NOESY NMR spectra) δ = 7.55 (d, J = 8.2 Hz, 6H; H_c), 7.51 (d, J = 8.3 Hz, 6H; H_d), 7.45 (d, J = 8.2 Hz, 6H; H_b), 7.31 (d, J = 8.3 Hz, 6H; H_e), 7.17 (d, J = 8.6 Hz, 2H; H_f), 6.77 (d, J = 8.6 Hz, 2H; H_g), 4.72 (br. s, 1H; H_h), 1.35 (s, 27H; H_a) ppm; ^{13}C{^{1}H} NMR (101 MHz, $CDCl_3$) δ = 153.7, 150.3, 145.9, 139.4, 138.5, 137.8, 132.6, 131.6, 126.7, 126.1, 125.8, 114.5, 63.8, 34.7, 31.5 ppm; IR (ATR) ν = 3541 (br. very w), 3027 (very w), 2960 (m), 2901 (w), 2864 (w), 1610 (w), 1494 (s), 1392 (w), 1363 (m), 1268 (m), 1178 (m), 1112 (m), 1004 (m), 815 (s), 764 cm^{-1} (m).

M.p., ^{1}H NMR, ^{13}C NMR and IR spectral data are in agreement with those reported in literature.[1]

2-(4-(tris(4'-(*tert*-butyl)-[1,1'-biphenyl]-4-yl)methyl)phenoxy)acetonitrile (3):

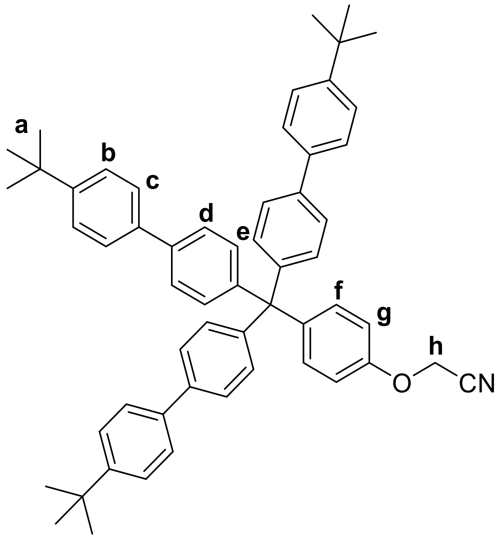

Chloroacetonitrile (0.23 mL, 3.6 mmol, 4 eq.) was added to compound **2** (0.66 g, 0.90 mmol) and K_2CO_3 (0.25 g, 1.8 mmol, 2 eq.) suspended in a mixture of acetone/CH_2Cl_2 3:1 (10 mL) under an argon atmosphere. The reaction mixture was heated to 50 °C and stirred for 5 days at this temperature. The resulting suspension was allowed to cool to rt and diluted with EtOAc (50 mL), after which the organic layer was washed with water (3 × 50 mL) and brine (25 mL), dried over anhydrous Na_2SO_4 and concentrated. Purification by column chromatography (SiO_2; CH_2Cl_2/pentane 1:1) afforded **3** as a white solid (0.50 g, 72%); R_f = 0.80 (SiO_2; CH_2Cl_2/pentane 1:1); m.p. 297 – 298 °C; ^{1}H NMR (400 MHz, $CDCl_3$; assignments are based on COSY and NOESY NMR spectra) δ = 7.55 (d, J = 8.4 Hz, 6H; H_c), 7.52 (d, J = 8.3 Hz, 6H; H_d), 7.46 (d, J = 8.4 Hz, 6H; H_b), 7.34 – 7.28 (m, 8H; H_e, H_f), 6.92 (d, J = 8.9 Hz, 2H; H_g), 4.77 (s, 2H; H_h), 1.36 (s, 27H; H_a) ppm; ^{13}C{^{1}H} NMR (101 MHz, $CDCl_3$) δ = 154.7, 150.4, 145.6, 141.8, 138.7, 137.8, 132.7, 131.5, 126.7, 126.2, 125.8, 115.3, 113.9, 63.8, 53.7, 34.7, 31.5 ppm; IR (ATR) ν = 3028 (very w), 2960 (m), 2901 (w), 2864 (w), 1604 (w), 1583 (very w), 1494 (s), 1461 (w), 1392 (w), 1362 (m), 1268 (m), 1220 (m), 1186 (m), 1113 (m), 1049 (m), 1004 (s), 815 (s), 764 (m), 746 (w), 732 cm^{-1} (w).

2-(4-(tris(4'-(*tert*-butyl)-[1,1'-biphenyl]-4-yl)methyl)phenoxy)ethan-1-amine (4):

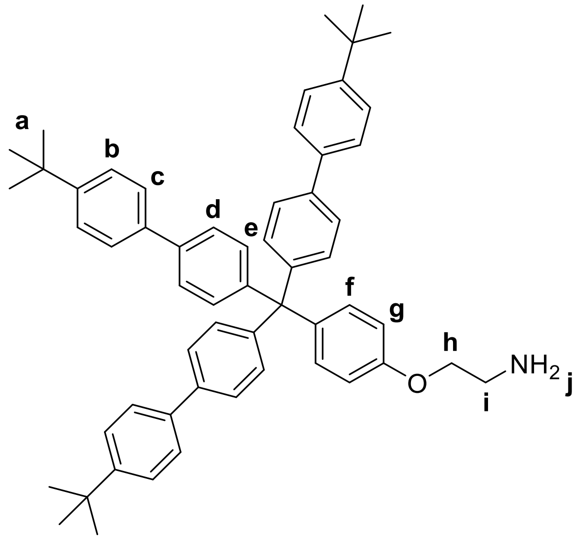

$LiAlH_4$ (1 M in THF, 1.3 mL, 1.3 mmol, 2 eq.) was added dropwise to compound **3** (0.50 g, 0.65 mmol) in dry THF (10 mL) under a nitrogen atmosphere at 0 °C and the reaction mixture was stirred for 30 min at this temperature. Then, it was treated with water (20 mL) and the aqueous phase was extracted with CH_2Cl_2 (5 × 40 mL). The combined organic layers were washed with brine (50 mL), dried over anhydrous Na_2SO_4, and concentrated to obtain **4** as an off-white solid (0.46 g, 91%) which was used without further purification in the next reaction step; R_f = 0.37 (SiO_2; CH_2Cl_2/MeOH 90:10); m.p. 218 – 220 °C; ^1H NMR (400 MHz, $CDCl_3$; assignments are based on COSY and NOESY NMR spectra) δ = 7.55 (d, J = 8.4 Hz, 6H; H_c), 7.51 (d, J = 8.5 Hz, 6H; H_d), 7.45 (d, J = 8.4 Hz, 6H; H_b), 7.31 (d, J = 8.5 Hz, 6H; H_e), 7.21 (d, J = 8.9 Hz, 2H; H_f), 6.84 (d, J = 8.9 Hz, 2H; H_g), 3.99 (t, J = 5.1 Hz, 2H; H_h), 3.08 (t, J = 5.1 Hz, 2H; H_i), 1.35 (s, 27H; H_a) ppm; $^{13}C\{^1H\}$ NMR (101 MHz, $CDCl_3$) δ = 156.7, 150.3, 145.9, 139.6, 138.5, 137.8, 132.4, 131.6, 126.7, 126.1, 125.8, 113.6, 68.3, 63.8, 41.1, 34.7, 31.5 ppm; IR (ATR) ν = 3387 (br. very w), 3027 (very w), 2960 (m), 2901 (w), 2867 (w), 1605 (w), 1494 (s), 1460 (m), 1392 (m), 1362 (m), 1245 (m), 1182 (m), 1113 (m), 1004 (s), 814 (s), 764 cm^{-1} (m); HRMS (ESI) m/z: 776.4818 ($[M+H]^+$, calcd for $C_{57}H_{62}NO^+$: 776.4826).

N[3],N[5]-bis(2-(4-(tris(4'-(*tert*-butyl)-[1,1'-biphenyl]-4-yl)methyl)phenoxy)ethyl)pyridine-3,5-dicarboxamide (5):

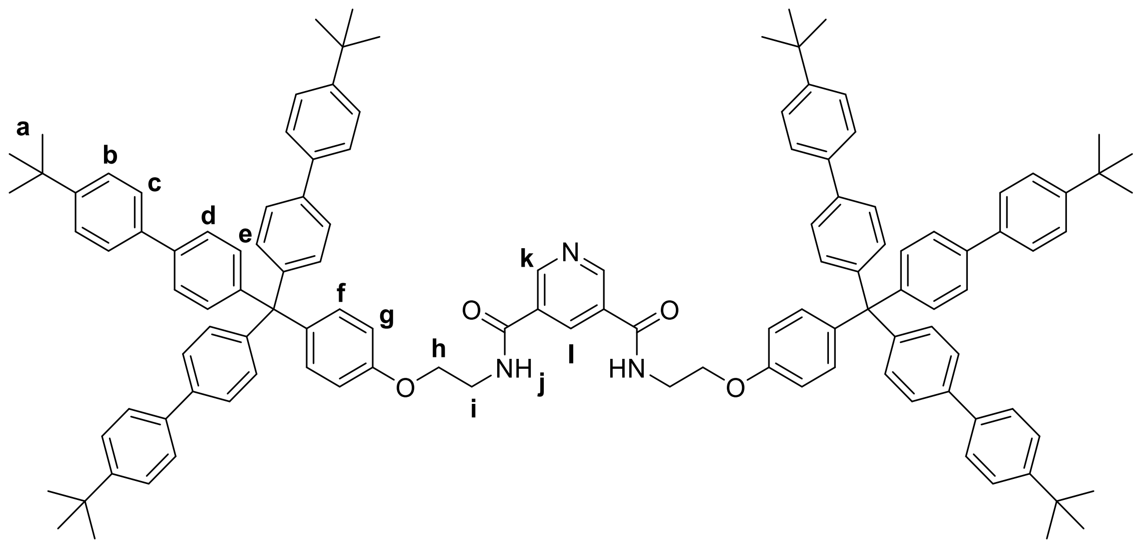

Compound **4** (0.42 g, 0.54 mmol, 2 eq.) was suspended in dry DMF (2 mL) under a nitrogen atmosphere. Then, Et_3N (0.11 mL, 0.79 mmol, 3 eq.) was added, followed by the dropwise addition of a solution of pyridine-3,5-dicarbonyl dichloride (54 mg, 0.26 mmol) in dry DMF (0.26 mL). The reaction mixture was subsequently heated to 50 °C and stirred for 18 h, after which it was allowed to cool to rt and diluted with EtOAc (40 mL). The organic layer was washed with water (5 × 40 mL) and brine (20 mL), dried over anhydrous Na_2SO_4, and concentrated. Purification by column chromatography (SiO_2; CH_2Cl_2/EtOAc 100:0 to 60:40) afforded **5** as an off-white solid (0.17 g, 37%); R_f = 0.61 (SiO_2; CH_2Cl_2/EtOAc 1:1); m.p. 250 °C; ^{1}H NMR (600 MHz, $CDCl_3$; assignments are based on COSY and NOESY NMR spectra) δ = 9.40 (s, 2H; H_k), 8.92 (s, 1H; H_l), 7.51 (d, J = 8.5 Hz, 12H; H_c), 7.47 (d, J = 8.5 Hz, 12H; H_d), 7.42 (d, J = 8.5 Hz, 12H; H_b), 7.28 (d, J = 8.5 Hz, 12H; H_e), 7.21 (d, J = 8.9 Hz, 4H; H_f), 6.81 (d, J = 8.9 Hz, 4H; H_g), 4.15 (t, J = 4.9 Hz, 4H; H_h), 3.88 (q, J = 4.9 Hz, 4H; H_i), 1.33 (s, 54H; H_a) ppm; $^{13}C\{^1H\}$ NMR (151 MHz, $CDCl_3$) δ = 162.9, 156.4, 150.3, 147.8, 145.8, 139.9, 138.5, 137.8, 137.6, 132.5, 131.9, 131.5, 126.7, 126.1, 125.8, 113.5, 66.2, 63.8, 40.1, 34.6, 31.5 ppm; IR (ATR) ν = 3373 (br. very w), 3029 (very w), 2960 (m), 2904 (w), 2864 (w), 1671 (br. m), 1605 (w), 1494 (s), 1461 (m), 1392 (m), 1362 (m), 1269 (m), 1245 (m), 1183 (m), 1113 (m), 1033 (w), 1004 (s), 916 (w), 813 (s), 764 (m), 744 (m), 622 cm^{-1} (w).

1-methyl-3,5-bis((2-(4-(tris(4'-(*tert*-butyl)-[1,1'-biphenyl]-4-yl)methyl)phenoxy)ethyl)carbamoyl) pyridin-1-ium iodide (6·I):

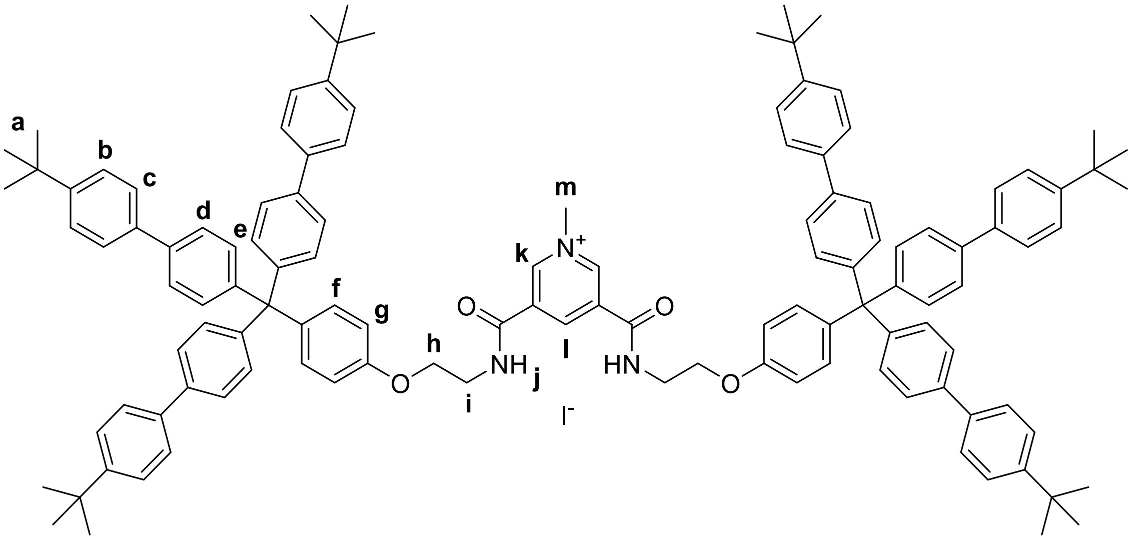

Compound **5** (64 mg, 38 µmol) was dissolved in iodomethane (2 mL) and stirred at rt for 17 h. The excess iodomethane was then distilled off under reduced pressure to give **6**·I as a yellow solid (63 mg, 91%) which was used without further purification in the next reaction step; R_f = 0.44 (SiO_2; CH_2Cl_2/MeOH 90:10); ^{1}H NMR (400 MHz, $CDCl_3$; assignments are based on COSY and NOESY NMR spectra) δ = 10.13 (br. s, 1H; H_l), 9.20 – 9.02 (m, 4H; H_j, H_k), 7.51 (d, J = 8.4 Hz, 12H; H_c), 7.46 (d, J = 8.4 Hz, 12H; H_d), 7.42 (d, J = 8.4 Hz, 12H; H_b), 7.29 (d, J = 8.4 Hz, 12H; H_e), 7.19 (d, J = 8.8 Hz, 4H; H_f), 6.85 (d, J = 8.8 Hz, 4H; H_g), 4.35 (s, 3H; H_m), 4.20 (t, J = 5.4 Hz, 4H; H_h), 3.89 (q, J = 5.4 Hz, 4H; H_i), 1.33 (s, 54H; H_a) ppm; ^{13}C{^{1}H} NMR (151 MHz, $CDCl_3$) δ = 160.8, 156.6, 150.3, 145.9, 144.1, 142.5, 139.6, 138.5, 137.8, 135.7, 132.4, 131.5, 126.7, 126.1, 125.8, 113.8, 65.8, 63.8, 49.5, 40.4, 34.6, 31.5 ppm; IR (ATR) ν = 3231 (br. very w), 3075 (br. w, sh), 3029 (w), 2962 (s), 2903 (w), 2868 (w), 1681 (m), 1606 (w), 1552 (w), 1495 (s), 1463 (m), 1393 (w), 1363 (w), 1249 (m), 1186 (m), 1113 (w), 1056 (w), 1033 (w), 1004 (m), 912 (very w), 816 (s), 765 (w), 733 (w), 698 (very w), 672 (very w), 623 cm^{-1} (very w); HRMS (ESI) *m*/*z*: 1697.9774 ([M]$^+$, calcd for $C_{122}H_{126}N_3O_4^+$: 1697.9776).

1-methyl-3,5-bis((2-(4-(tris(4'-(*tert*-butyl)-[1,1'-biphenyl]-4-yl)methyl)phenoxy)ethyl)carbamoyl) pyridin-1-ium chloride (6·Cl):

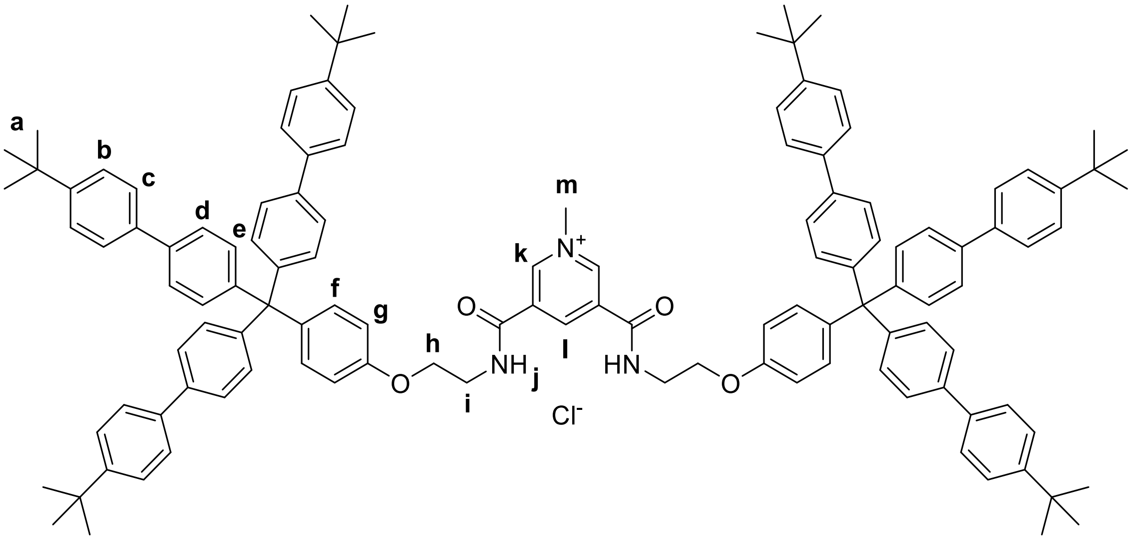

Compound **6**·I (60 mg, 33 µmol) was dissolved in $CHCl_3$ (40 mL) and the resulting solution was washed with 1 M NH_4Cl aq. (8 × 40 mL) and then with water (2 × 40 mL). The organic layer was dried over anhydrous Na_2SO_4 and concentrated to give **6**·Cl as a light yellow solid (57 mg, 100%) which was used without further purification in the next reaction step; R_f = 0.44 (SiO_2; CH_2Cl_2/MeOH 90:10); ^{1}H NMR (600 MHz, $CDCl_3$; assignments are based on COSY and NOESY NMR spectra) δ = 10.52 (s, 1H; H_l), 9.58 (t, J = 5.4 Hz, 2H; H_j), 9.15 (s, 2H; H_k), 7.50 (d, J = 8.5 Hz, 12H; H_c), 7.46 (d, J = 8.5 Hz, 12H; H_d), 7.41 (d, J = 8.5 Hz, 12H; H_b), 7.30 (d, J = 8.5 Hz, 12H; H_e), 7.20 (d, J = 8.9 Hz, 4H; H_f), 6.87 (d, J = 8.9 Hz, 4H; H_g), 4.25 – 4.14 (m, 7H; H_h, H_m), 3.85 (q, J = 5.4 Hz, 4H; H_i), 1.33 (s, 54H; H_a) ppm; ^{13}C{^{1}H} NMR (101 MHz, $CDCl_3$) δ = 160.6, 156.7, 150.3, 146.5, 145.9, 141.9, 139.7, 138.4, 137.7, 134.2, 132.3, 131.5, 126.7, 126.1, 125.8, 114.0, 66.0, 63.8, 49.3, 40.3, 34.6, 31.5 ppm; IR (ATR) ν = 3221 (br. very w), 3075 (w, sh), 3029 (w), 2962 (s), 2903 (w), 2868 (w), 1683 (m), 1606 (w), 1556 (w), 1495 (s), 1463 (m), 1393 (m), 1363 (m), 1291 (m, sh), 1269 (m, sh), 1249 (m), 1184 (m), 1113 (m), 1058 (very w), 1033 (very w), 1004 (m), 910 (w), 816 (s), 764 (w), 733 (m), 673 (very w), 637 (very w), 622 cm^{-1} (very w); HRMS (ESI) *m*/*z*: 1697.9782 ([M]$^+$, calcd for $C_{122}H_{126}N_3O_4^+$: 1697.9776).

NMR spectra of new compounds

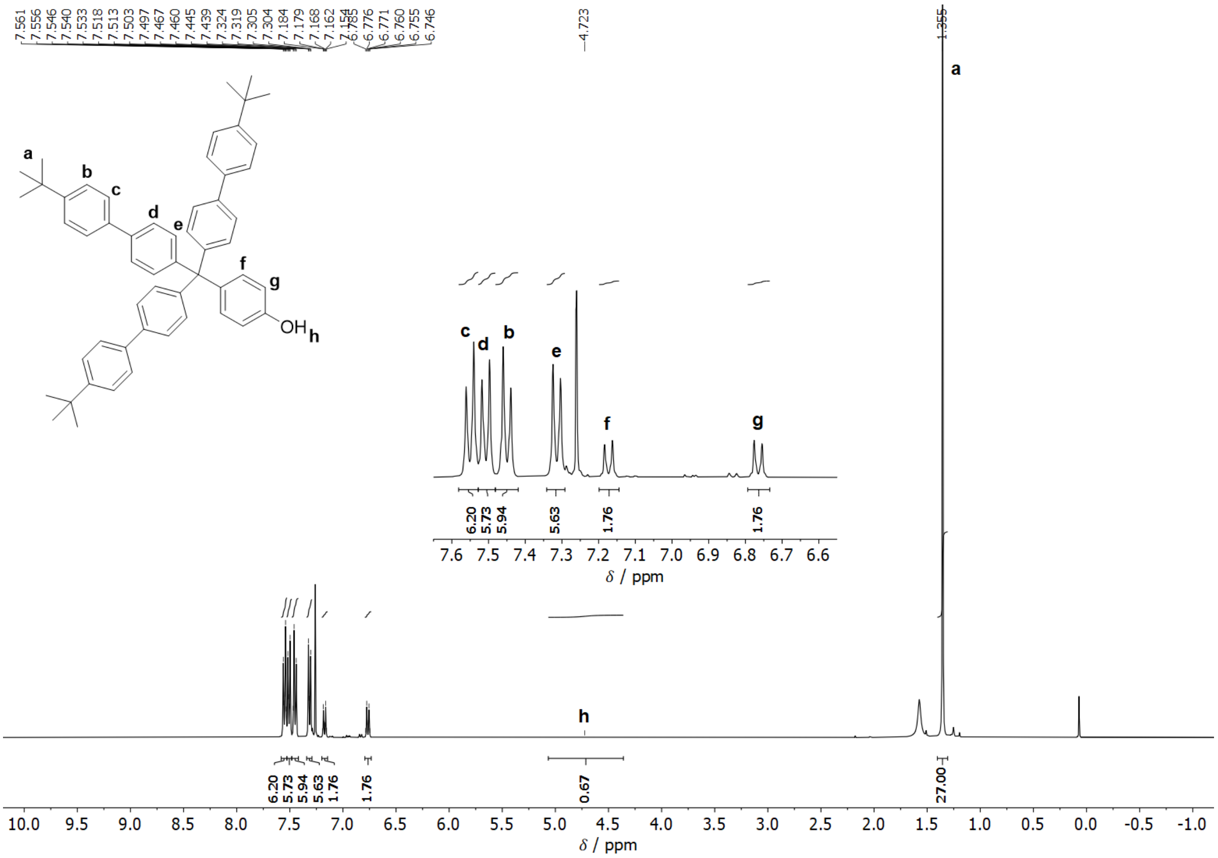

Figure S1: ^{1}H NMR (400 MHz, $CDCl_3$) of compound **2** measured at 294 K.

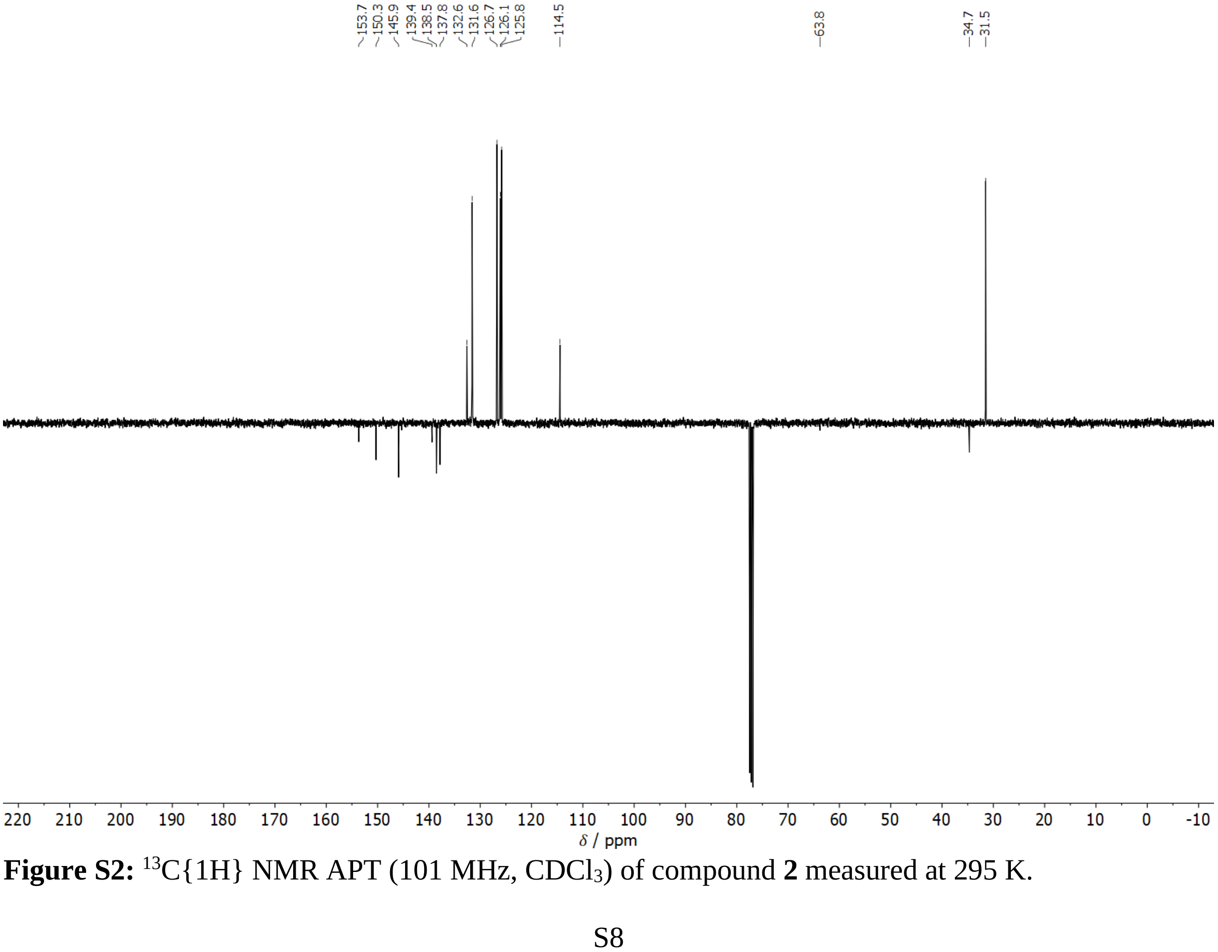

Figure S2: ^{13}C{1H} NMR APT (101 MHz, $CDCl_3$) of compound **2** measured at 295 K.

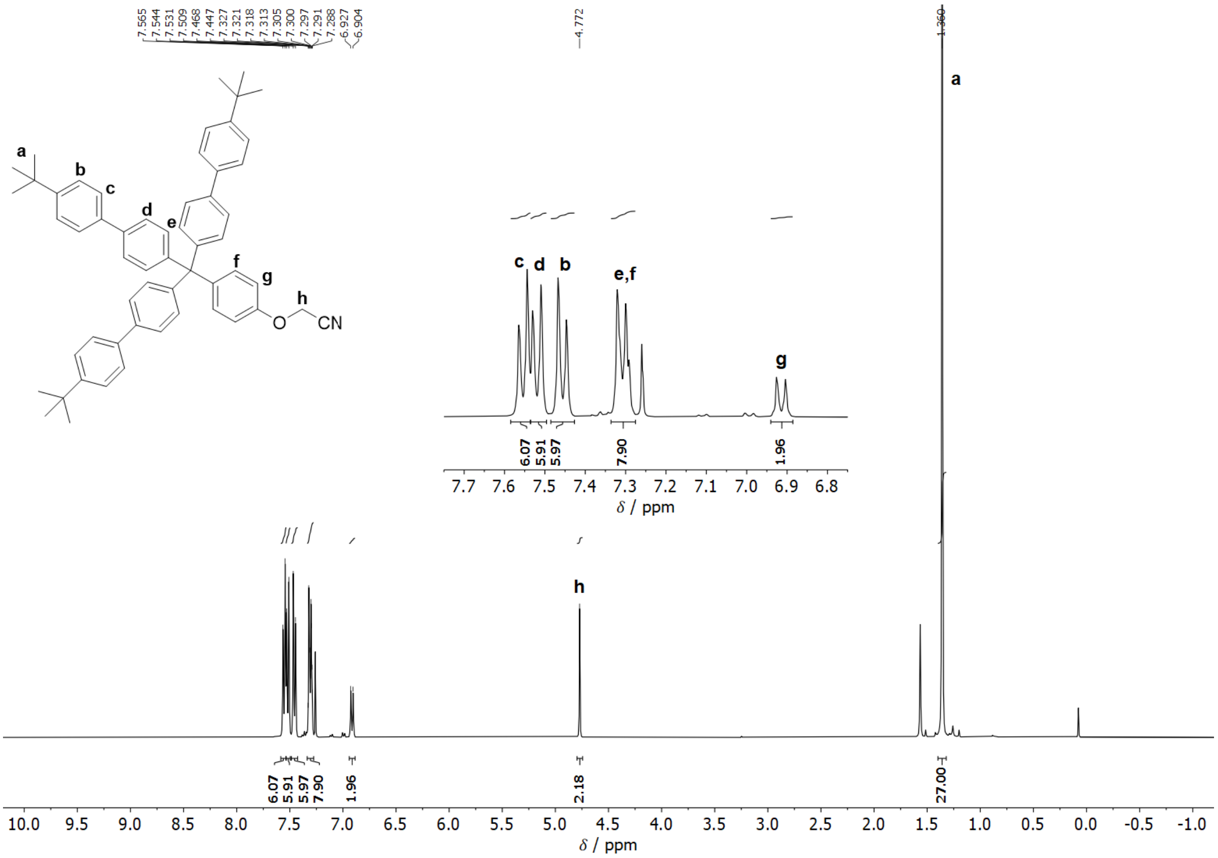

Figure S3: ^{1}H NMR (400 MHz, $CDCl_3$) of compound **3** measured at 294 K.

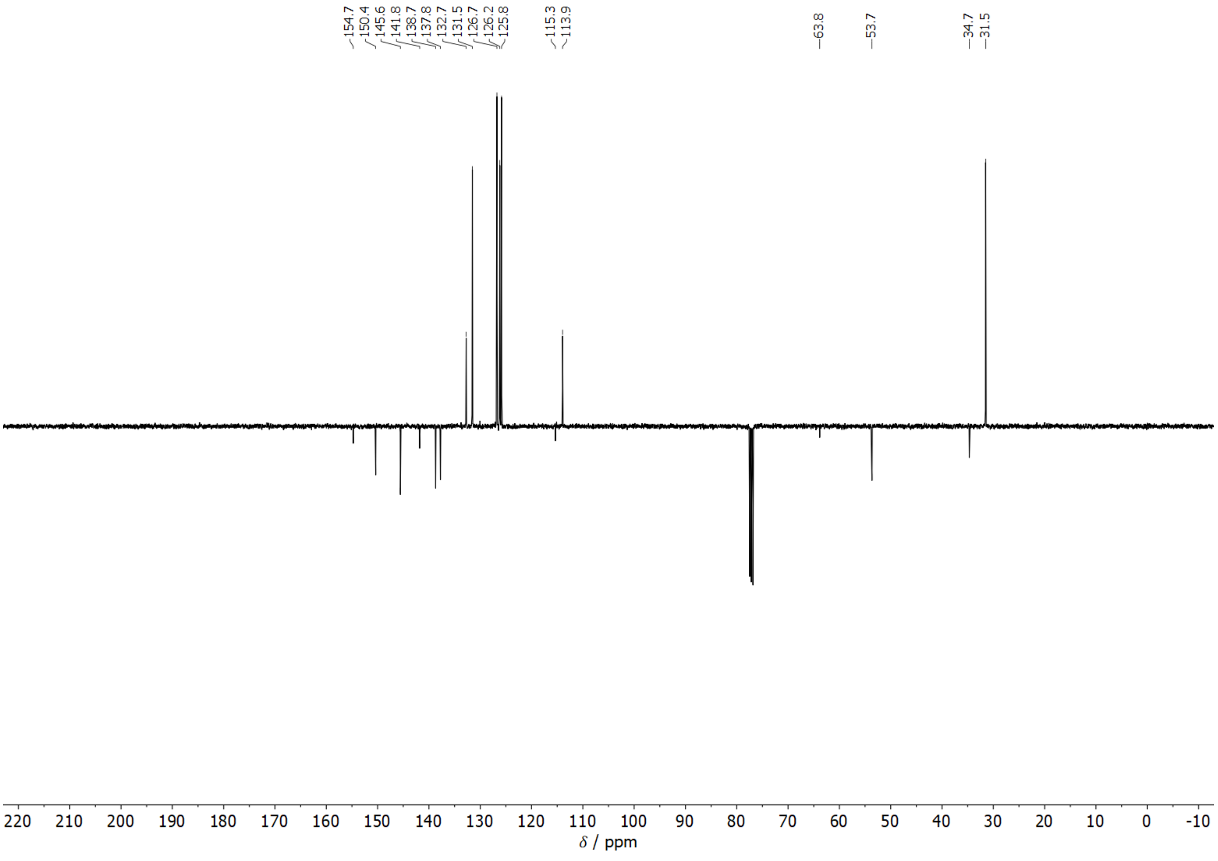

Figure S4: $^{13}C\{1H\}$ NMR APT (101 MHz, $CDCl_3$) of compound **3** measured at 295 K.

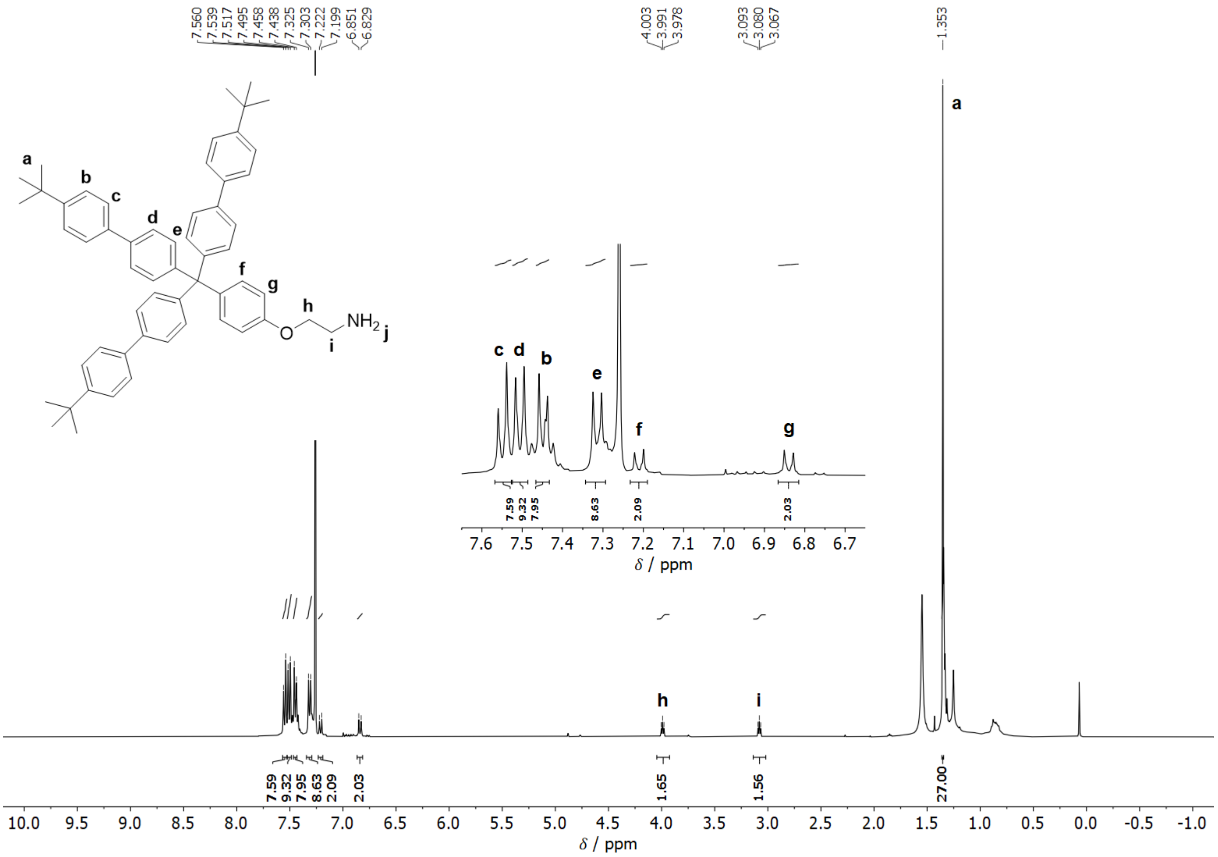

Figure S5: ^{1}H NMR (400 MHz, $CDCl_3$) of compound **4** measured at 293 K.

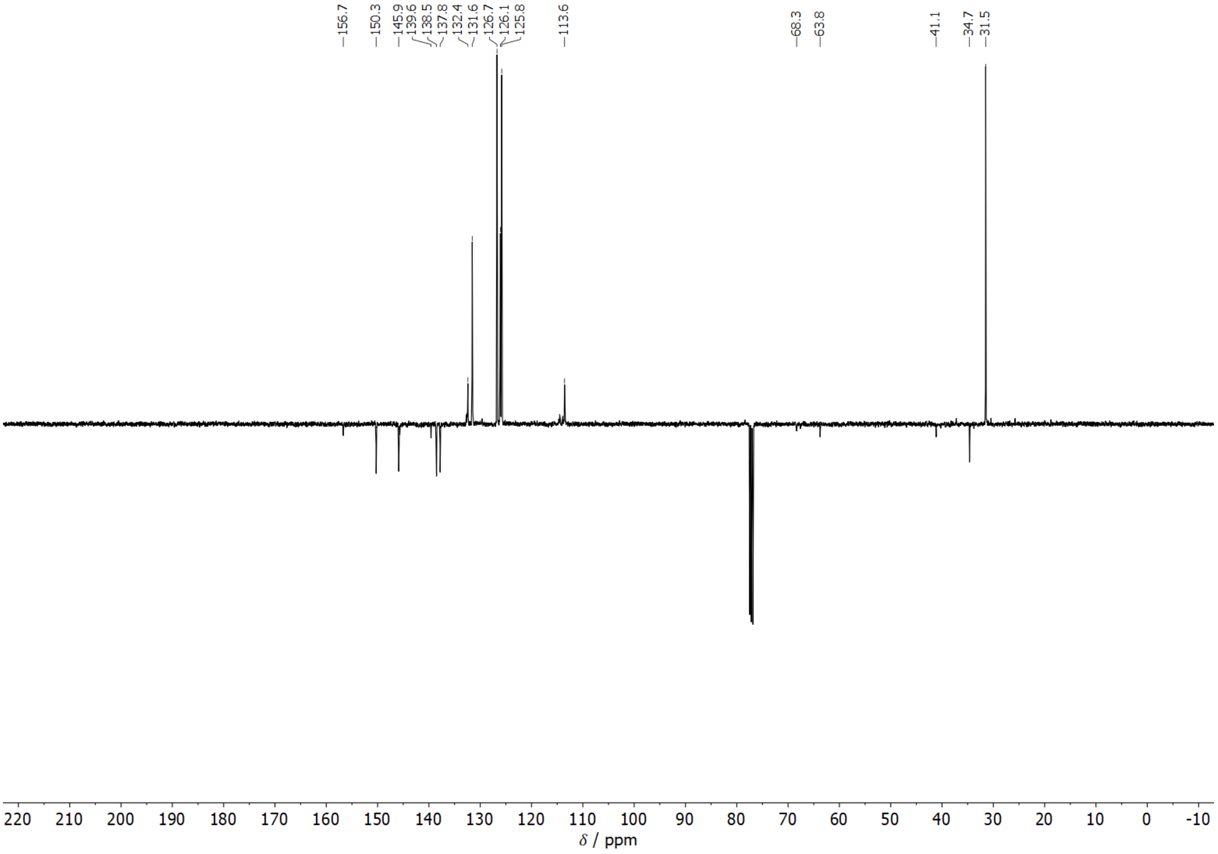

Figure S6: $^{13}C\{1H\}$ NMR APT (101 MHz, $CDCl_3$) of compound **4** measured at 295 K.

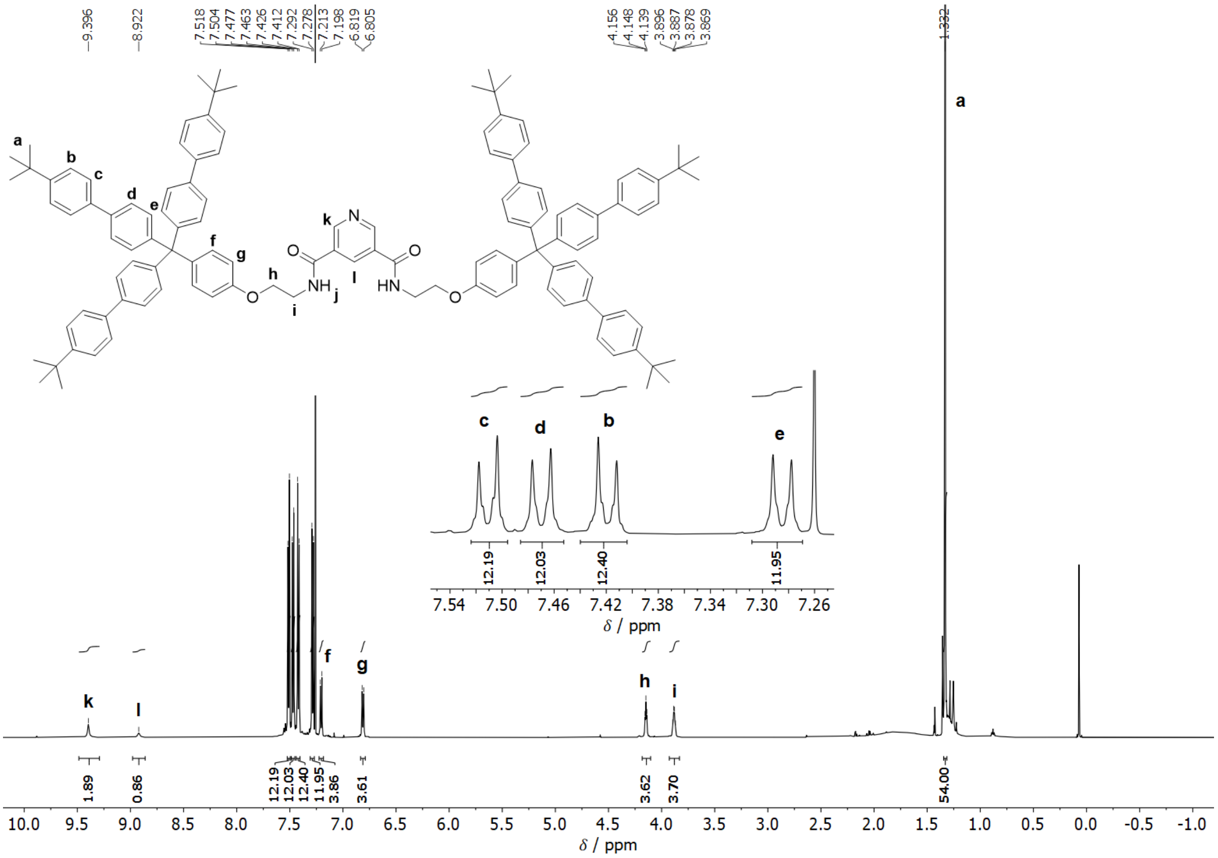

Figure S7: ^{1}H NMR (600 MHz, $CDCl_3$) of compound **5** measured at 298 K.

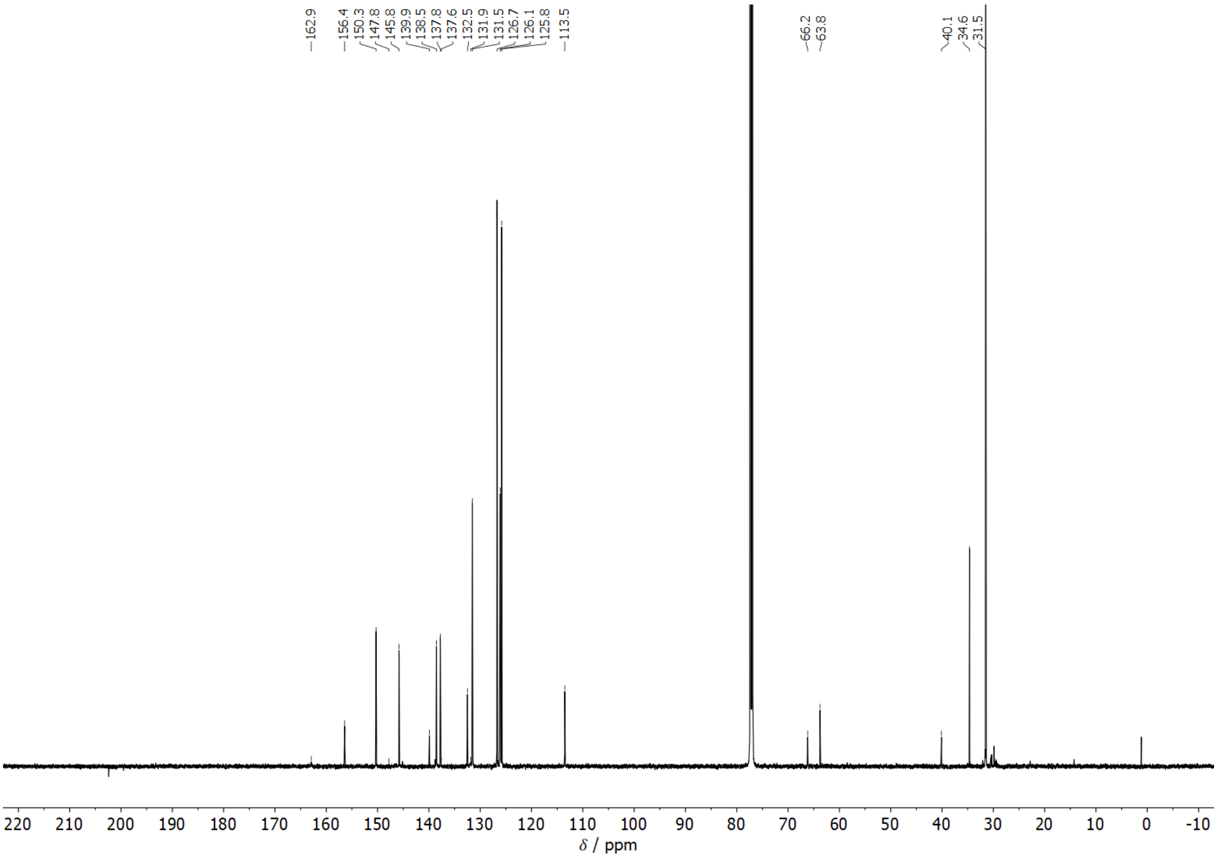

Figure S8: ^{13}C{1H} NMR (151 MHz, $CDCl_3$) of compound **5** measured at 298 K.

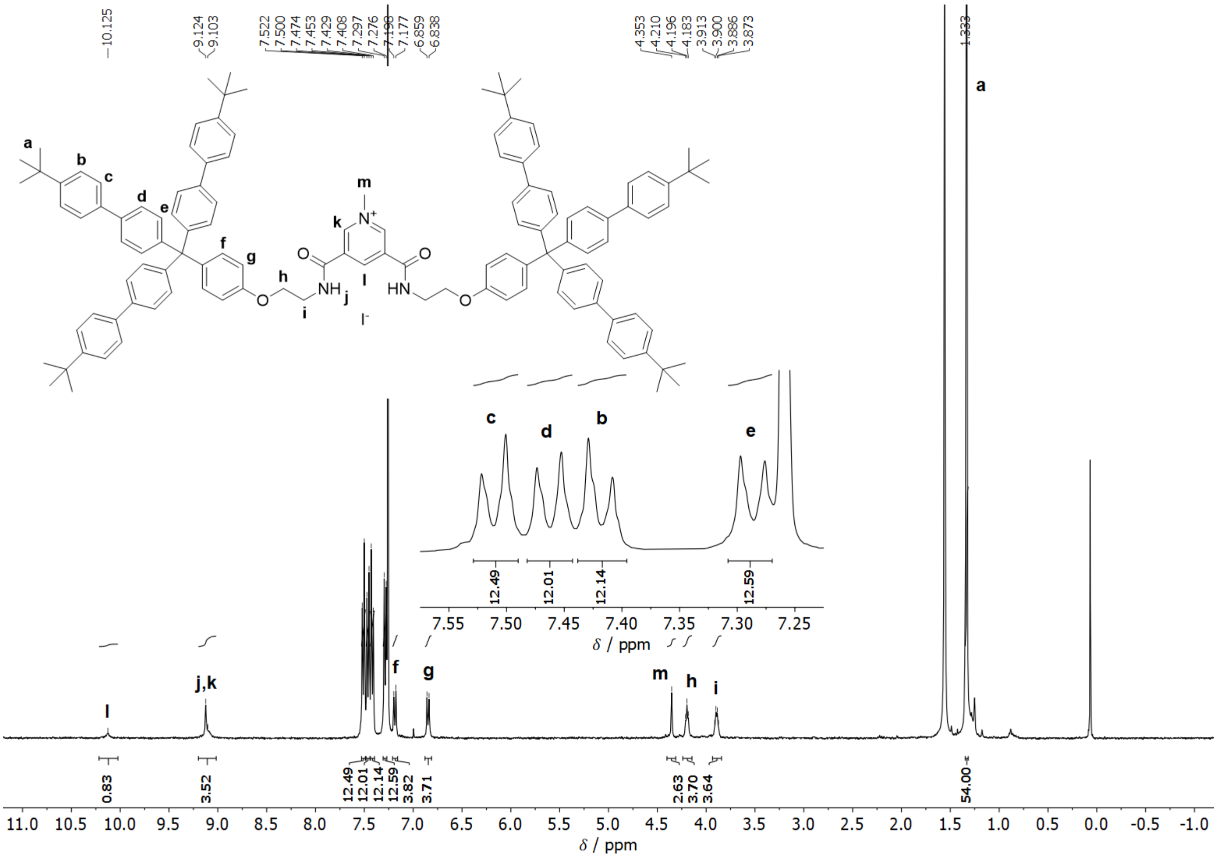

Figure S9: ^{1}H NMR (400 MHz, $CDCl_3$) of compound **6**·I measured at 294 K.

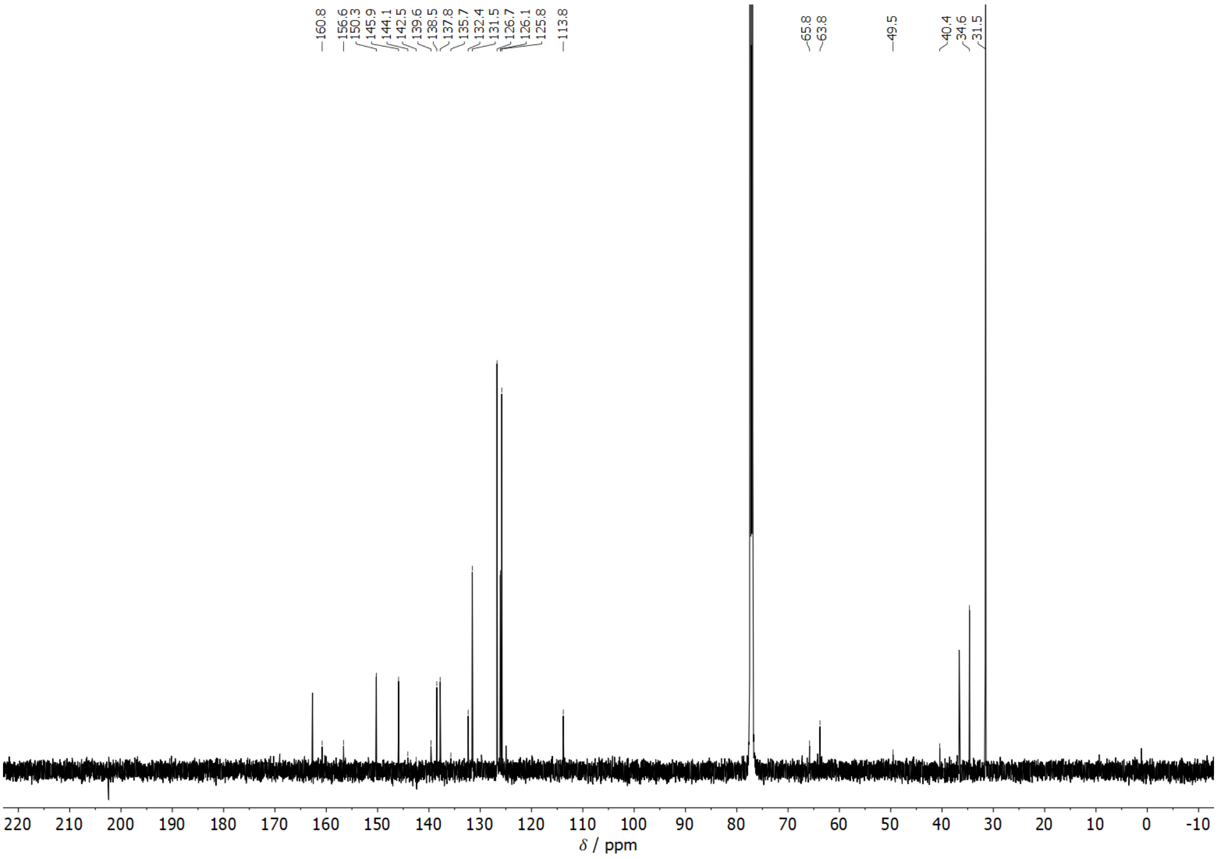

Figure S10: ^{13}C{1H} NMR (151 MHz, $CDCl_3$) of compound **6**·I measured at 298 K.

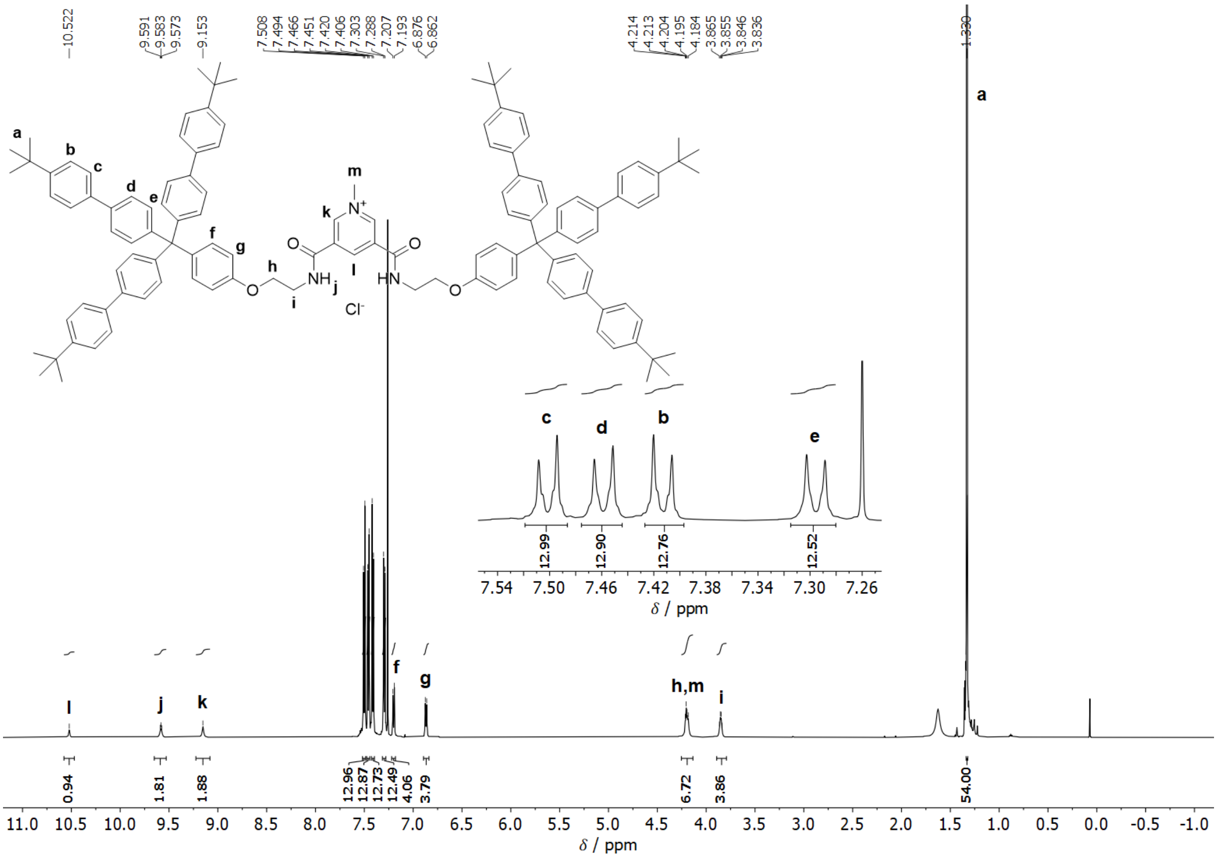

Figure S11: ^{1}H NMR (600 MHz, $CDCl_3$) of compound **6**·Cl measured at 298 K.

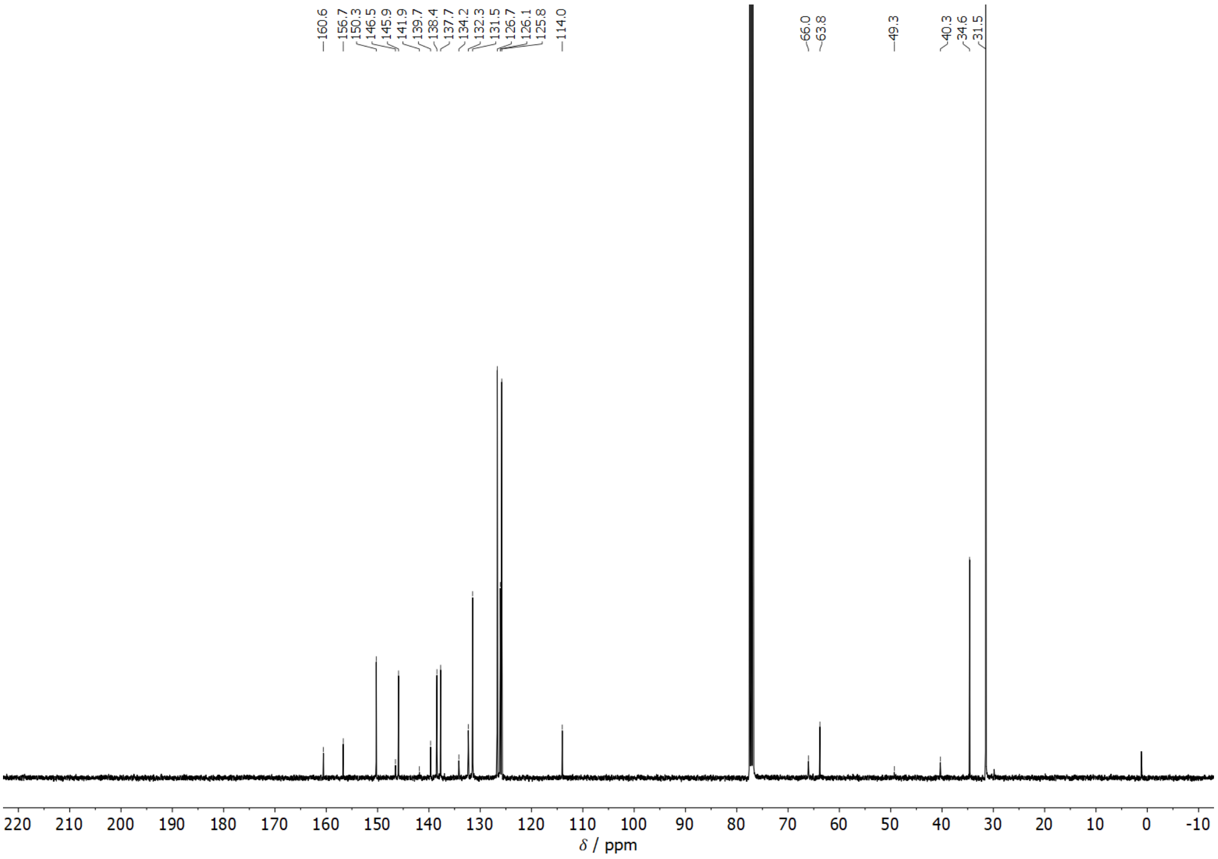

Figure S12: ^{13}C{1H} NMR (101 MHz, $CDCl_3$) of compound **6**·Cl measured at 295 K.

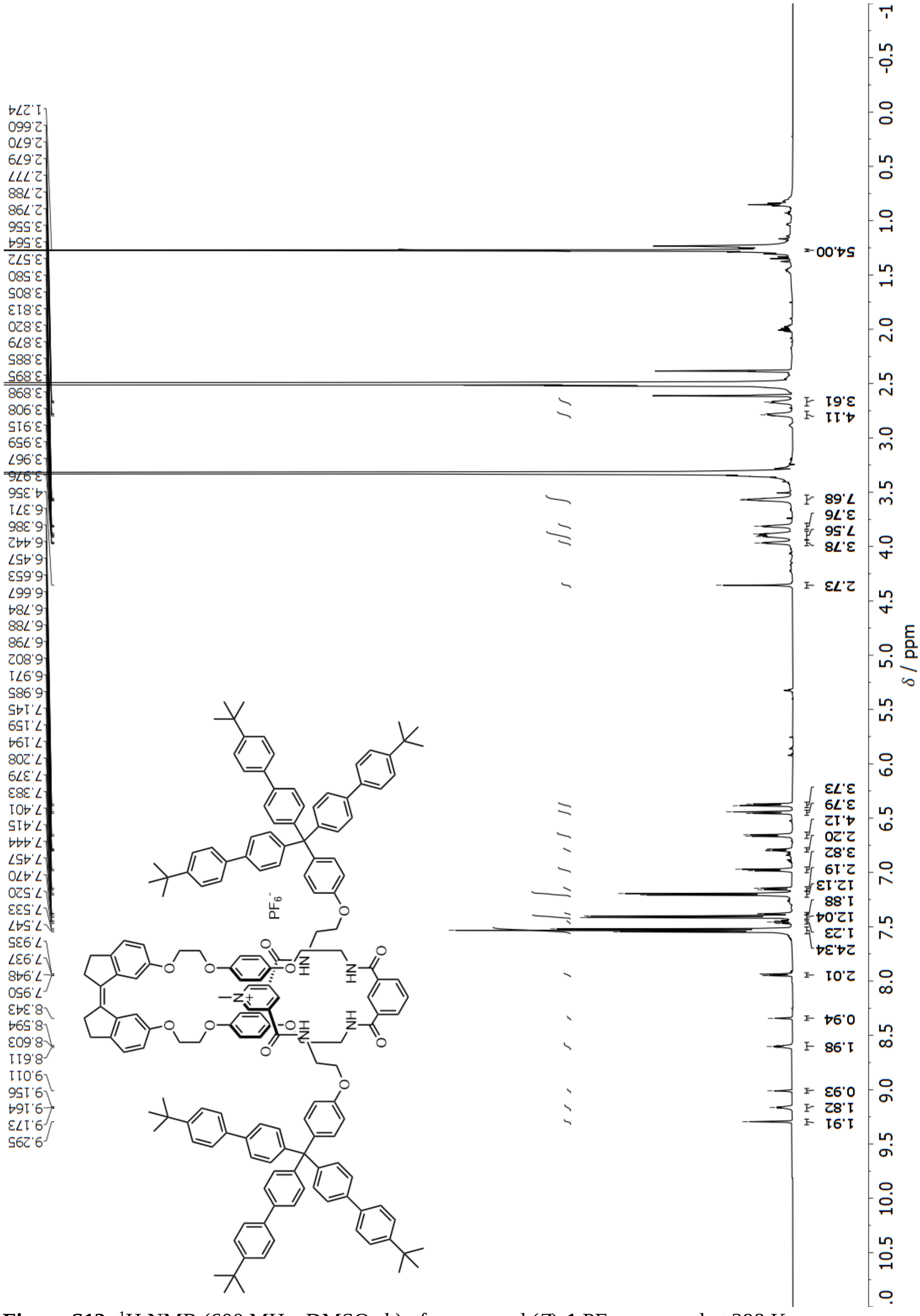

Figure S13: ^{1}H NMR (600 MHz, DMSO-d_6) of compound (*Z*)-**1**·PF_6 measured at 298 K.

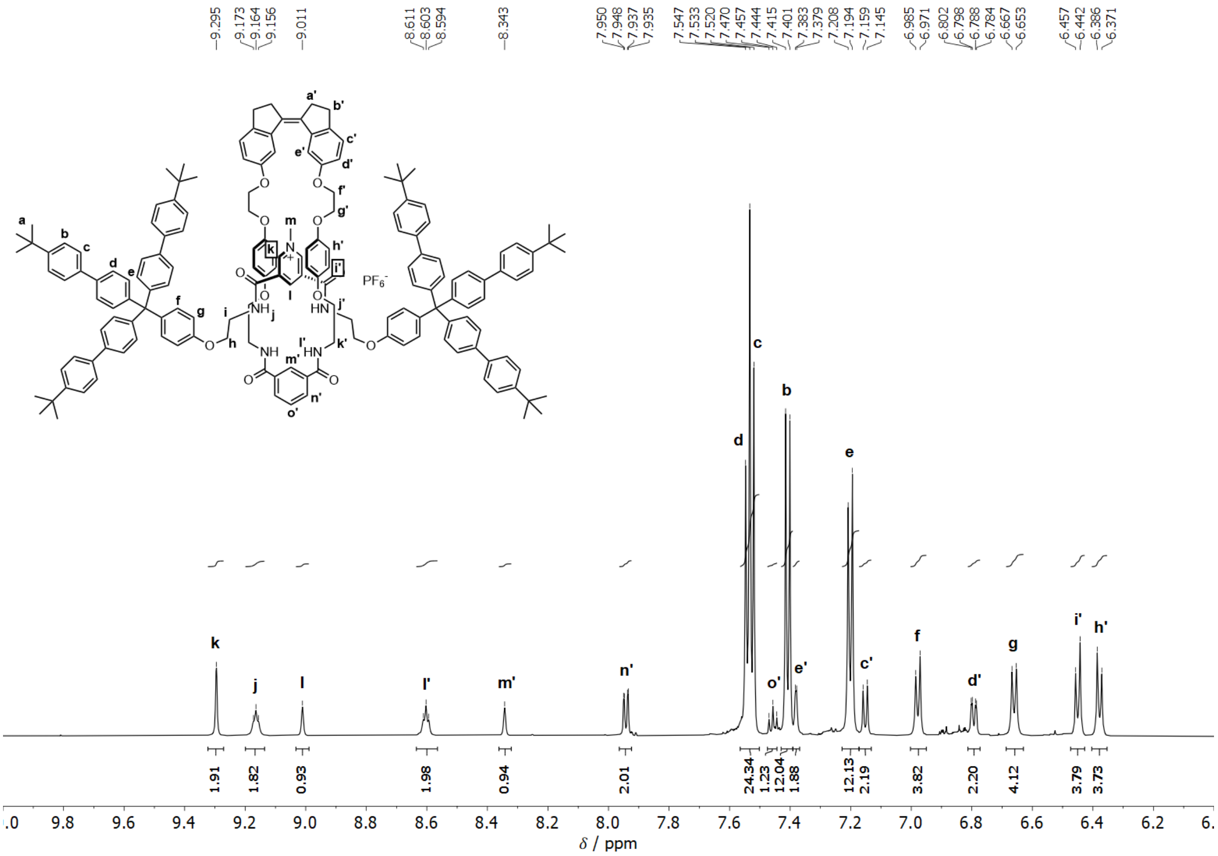

Figure S14: ^{1}H NMR (600 MHz, DMSO-d_6) of compound (Z)-**1**·PF_6 measured at 298 K, showing the downfield region of the spectrum including proton assignment.

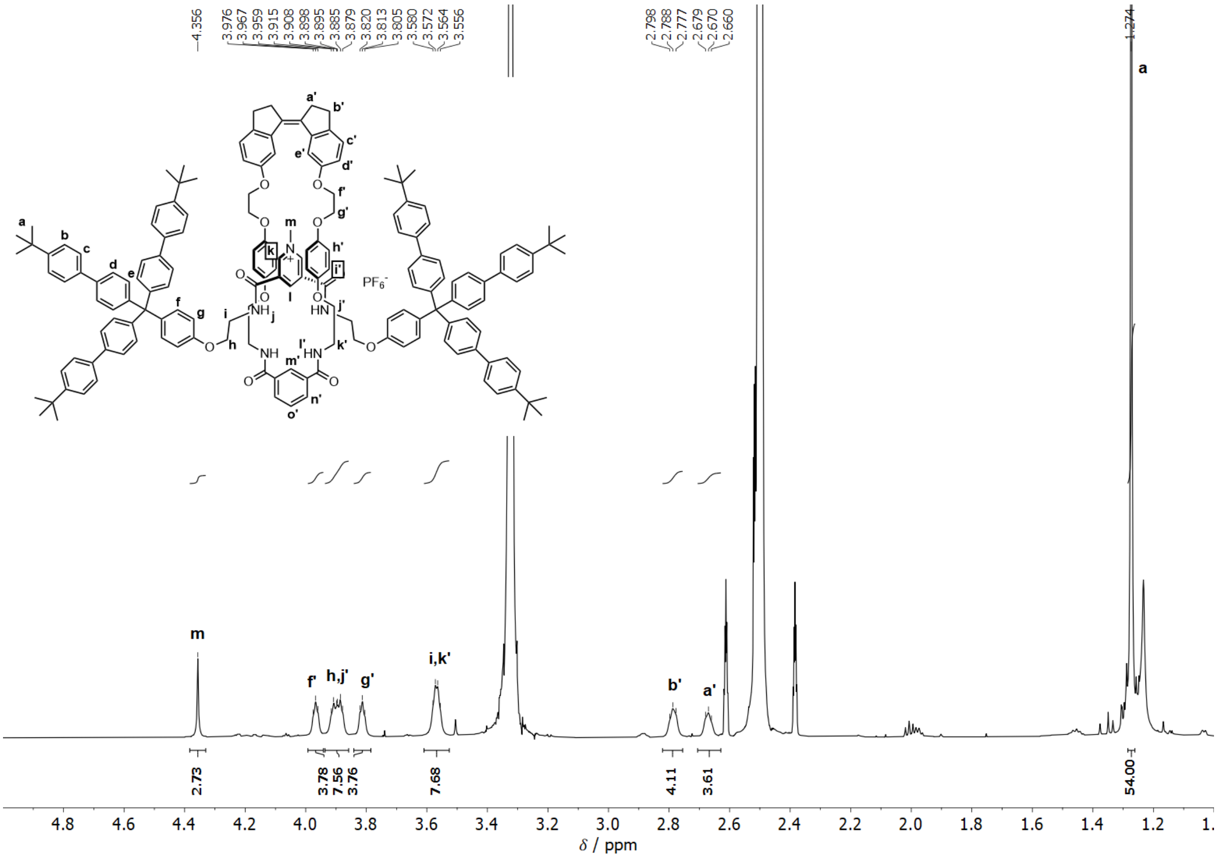

Figure S15: ^{1}H NMR (600 MHz, DMSO-d_6) of compound (Z)-**1**·PF_6 measured at 298 K, showing the upfield region of the spectrum including proton assignment.

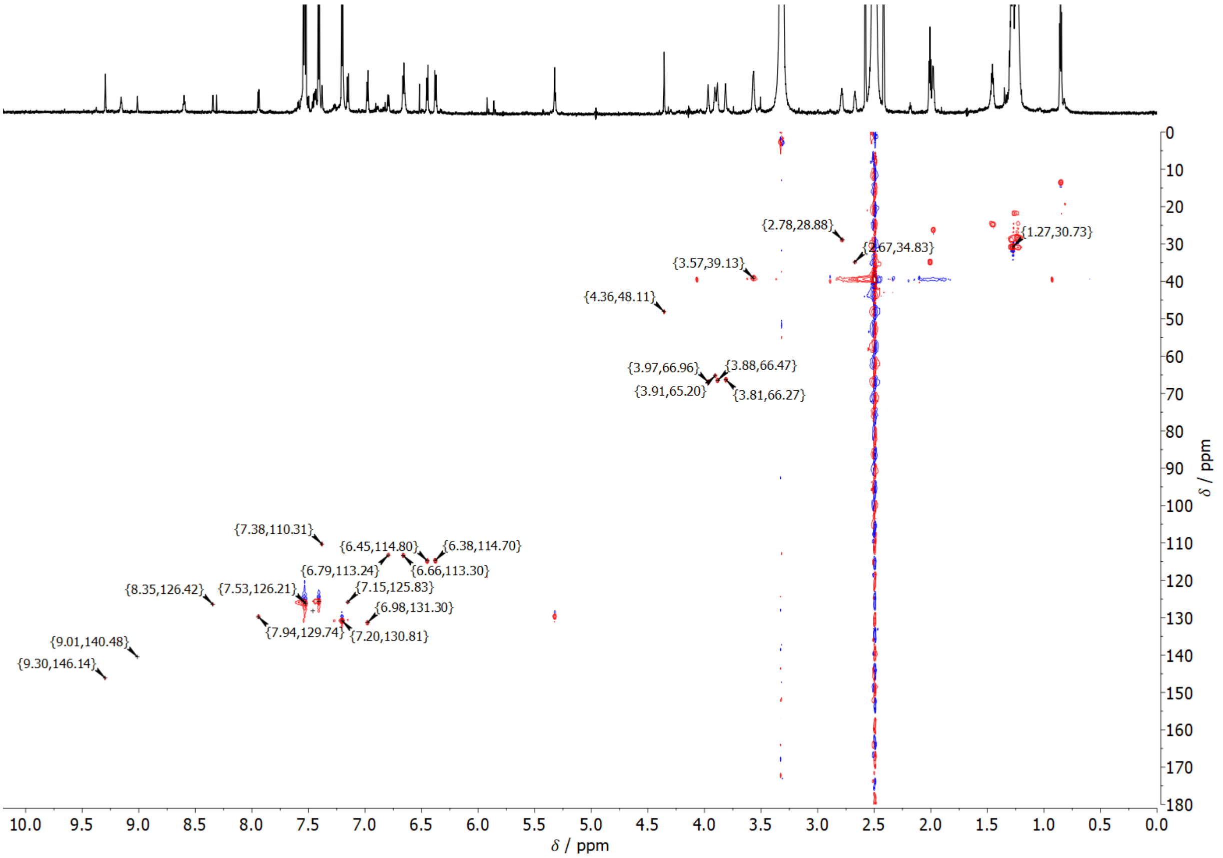

Figure S16: (^{1}H,^{13}C)-HSQC NMR (850/214 MHz, DMSO-d_6) of compound (*Z*)-**1**·PF_6 measured at 298 K.

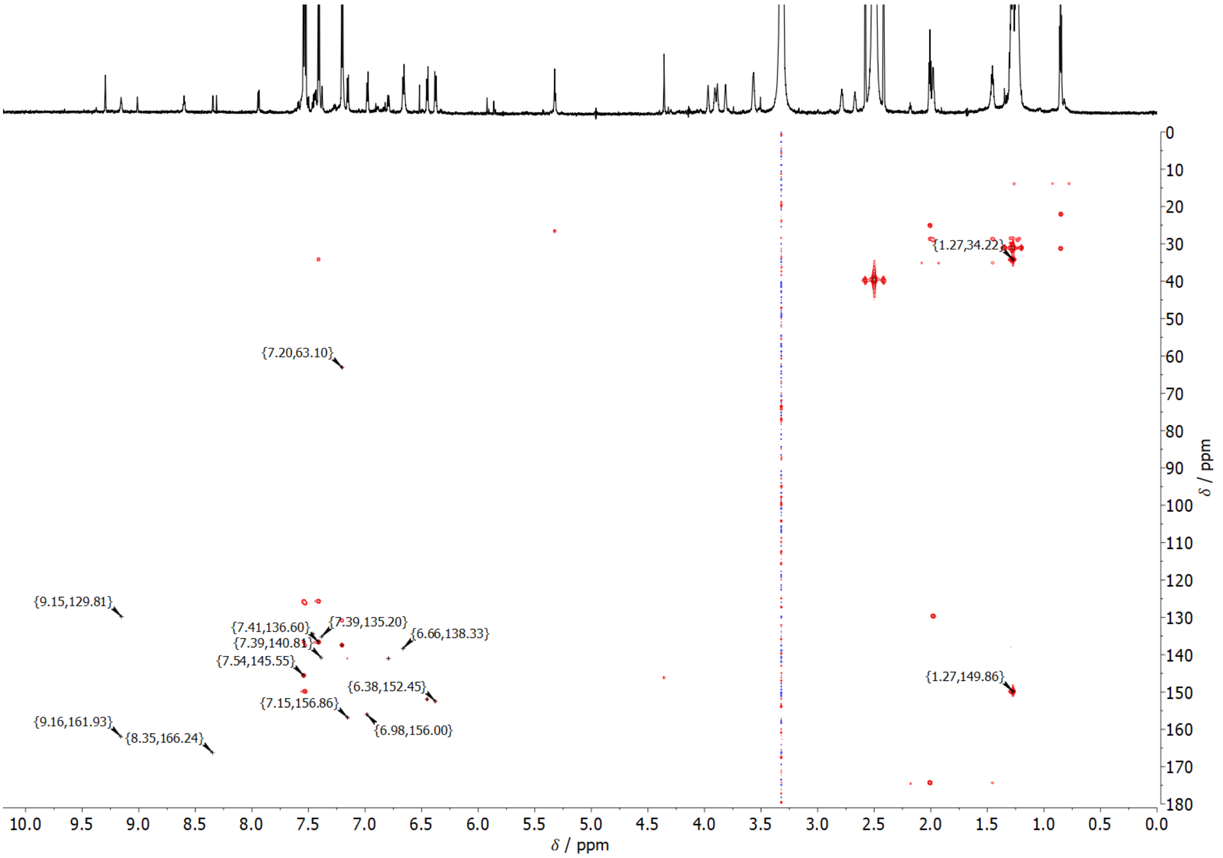

Figure S17: (^{1}H,^{13}C)-HMBC NMR (850/214 MHz, DMSO-d_6) of compound (*Z*)-**1**·PF_6 measured at 298 K.

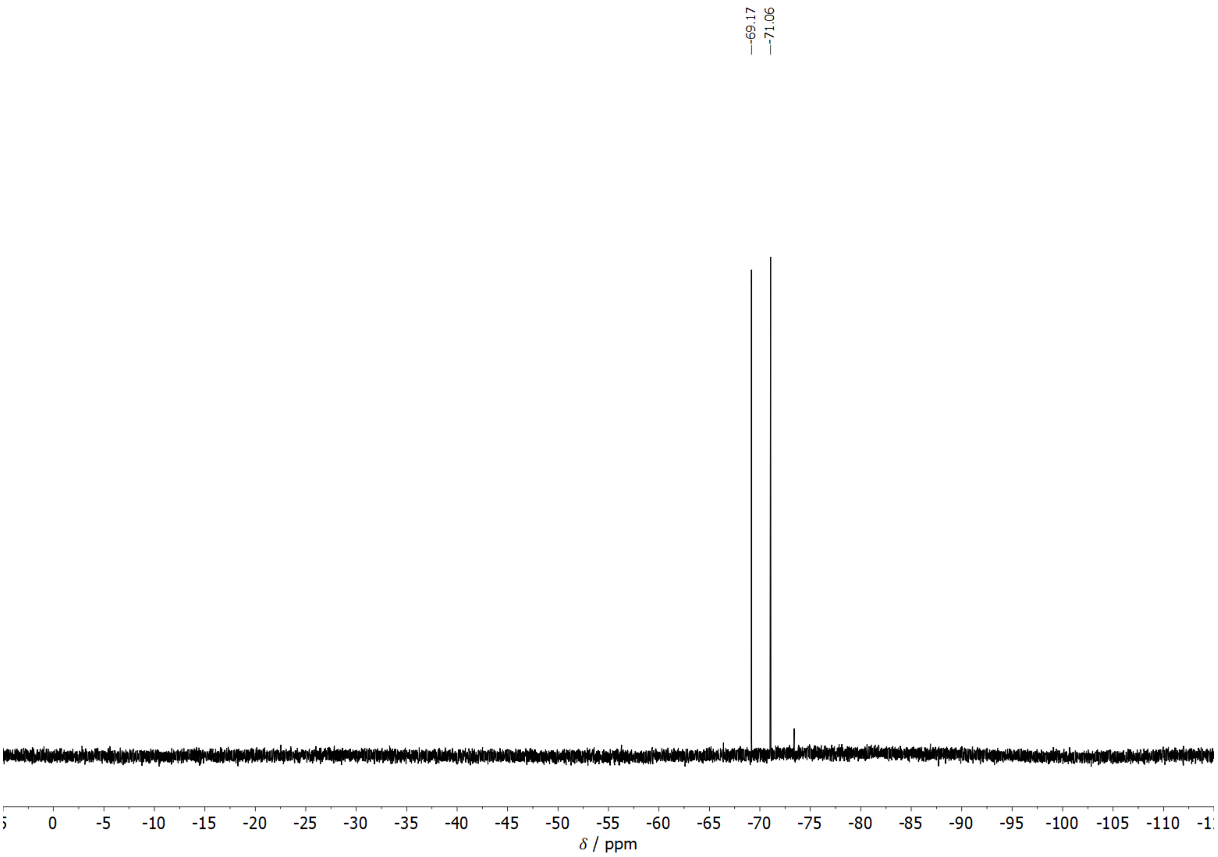

Figure S18: ^{19}F NMR (376 MHz, DMSO-d_6) of compound (Z)-**1**·PF_6 measured at 294 K.

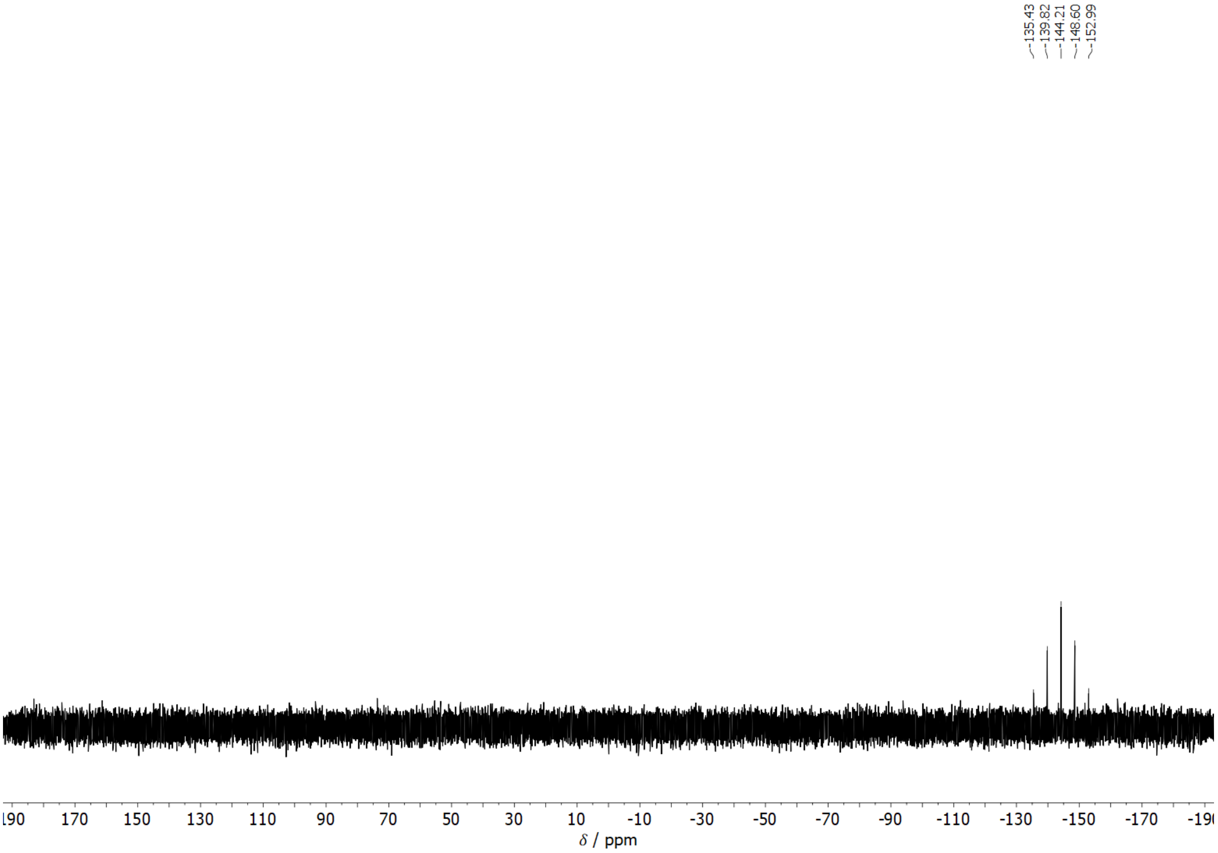

Figure S19: ^{31}P{^{1}H} NMR (162 MHz, DMSO-d_6) of compound (Z)-**1**·PF_6 measured at 295 K.

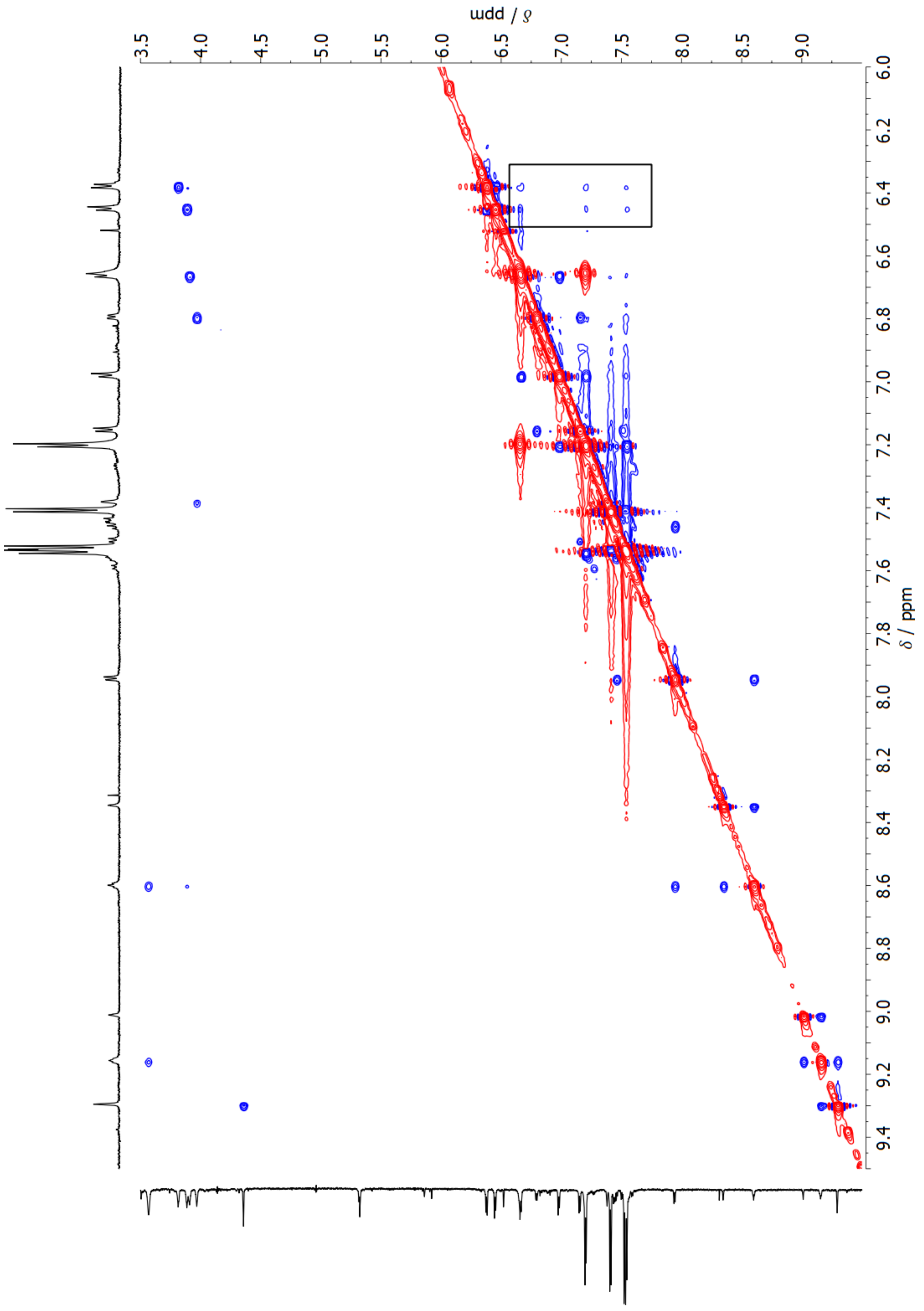

Figure S20: (^{1}H,^{1}H)-ROESY NMR (850 MHz, DMSO-d_6, τ_m = 200 ms) of compound (*Z*)-**1**·PF_6 measured at 298 K. The cross-peaks within the indicated box area confirm the mechanically interlocked nature of rotaxane (*Z*)-**1**·PF_6.

HRMS spectrum of title compound

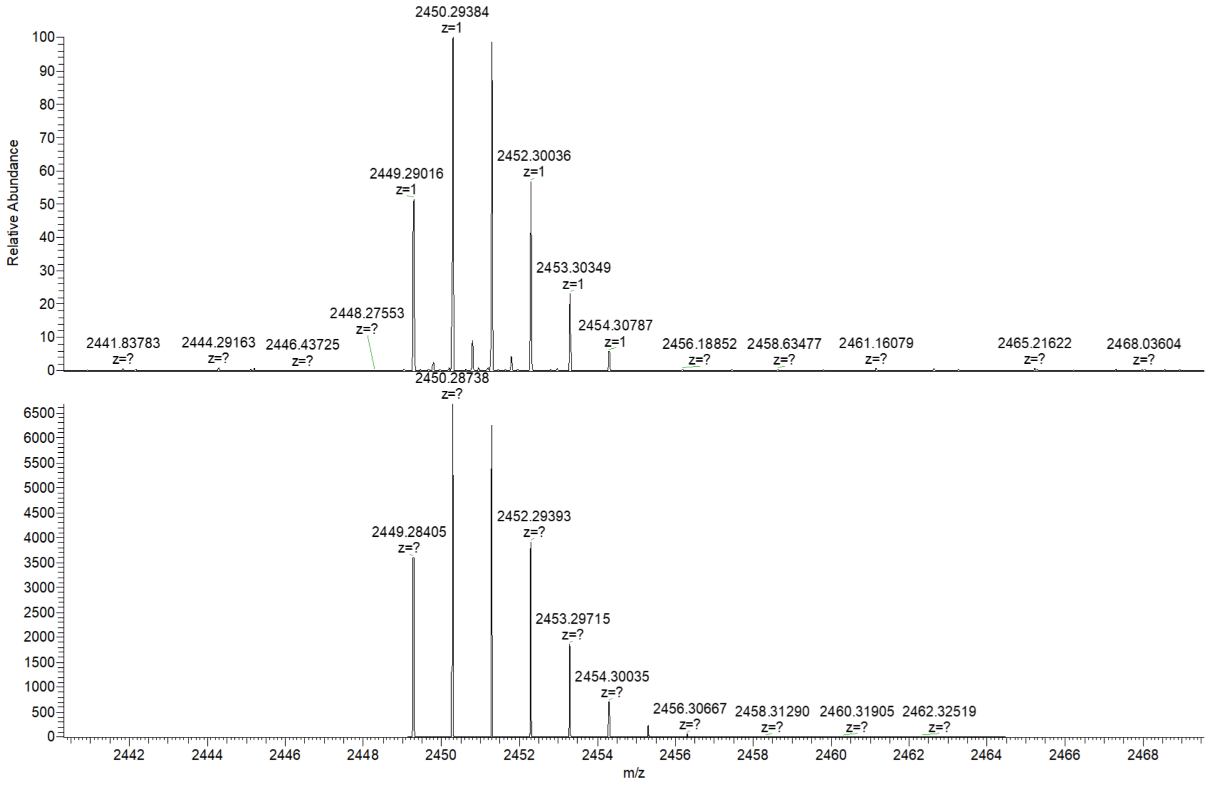

Figure S21. HRMS (ESI+) spectrum of (*Z*)-**1**·PF_6 showing the peak consistent with the positively charged fragment containing the interlocked macrocycle and axle without the hexafluorophosphate counterion (top) together with a simulated spectrum of the corresponding $C_{168}H_{170}N_5O_{12}^+$ fragment for reference (bottom).

UV-Vis irradiation experiments

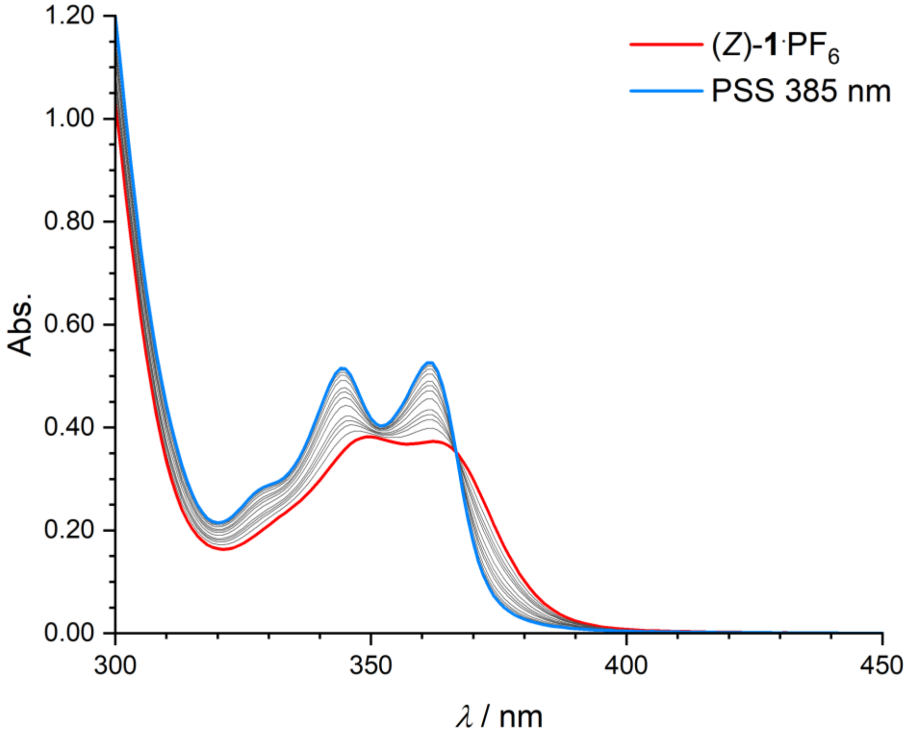

Figure S22: UV-Vis spectral changes of (*Z*)-**1**·PF_6 (2.0 × 10^{-5} M in dry and degassed DMSO) upon irradiation with 385 nm light for 160 s.

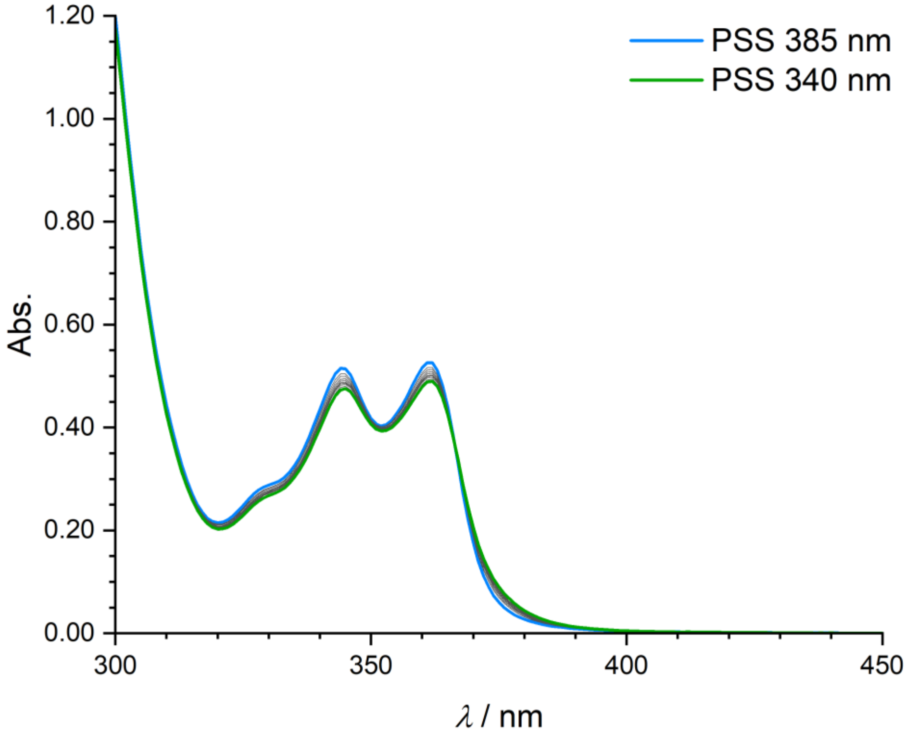

Figure S23: UV-Vis spectral changes of the PSS_{385} mixture of (*Z*)-**1**·PF_6 (2.0 × 10^{-5} M in dry and degassed DMSO) upon irradiation with 340 nm light for 120 s.

^{1}H NMR irradiation experiments

For the ^{1}H NMR irradiation experiment with (*Z*)-**1**·PF_6, a 0.20×10^{-3} M solution was prepared in 0.45 mL DMSO-d_6 that was purged with argon for 15 min.

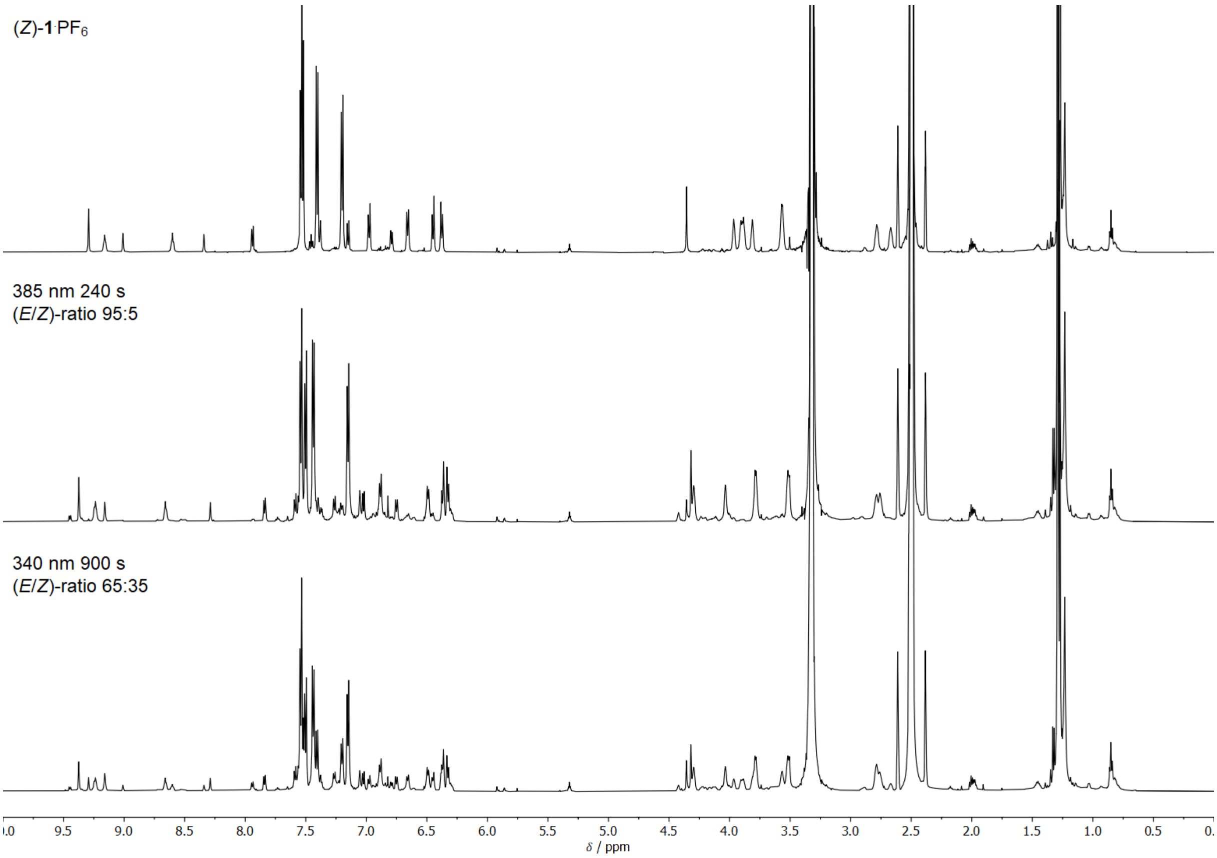

Figure S24: ^{1}H NMR spectral changes (600 MHz, 298 K) of (*Z*)-**1**·PF_6 (0.20 mM in DMSO-d_6) upon irradiation with 385 nm light for 240 s, followed by 340 nm light for 900 s.

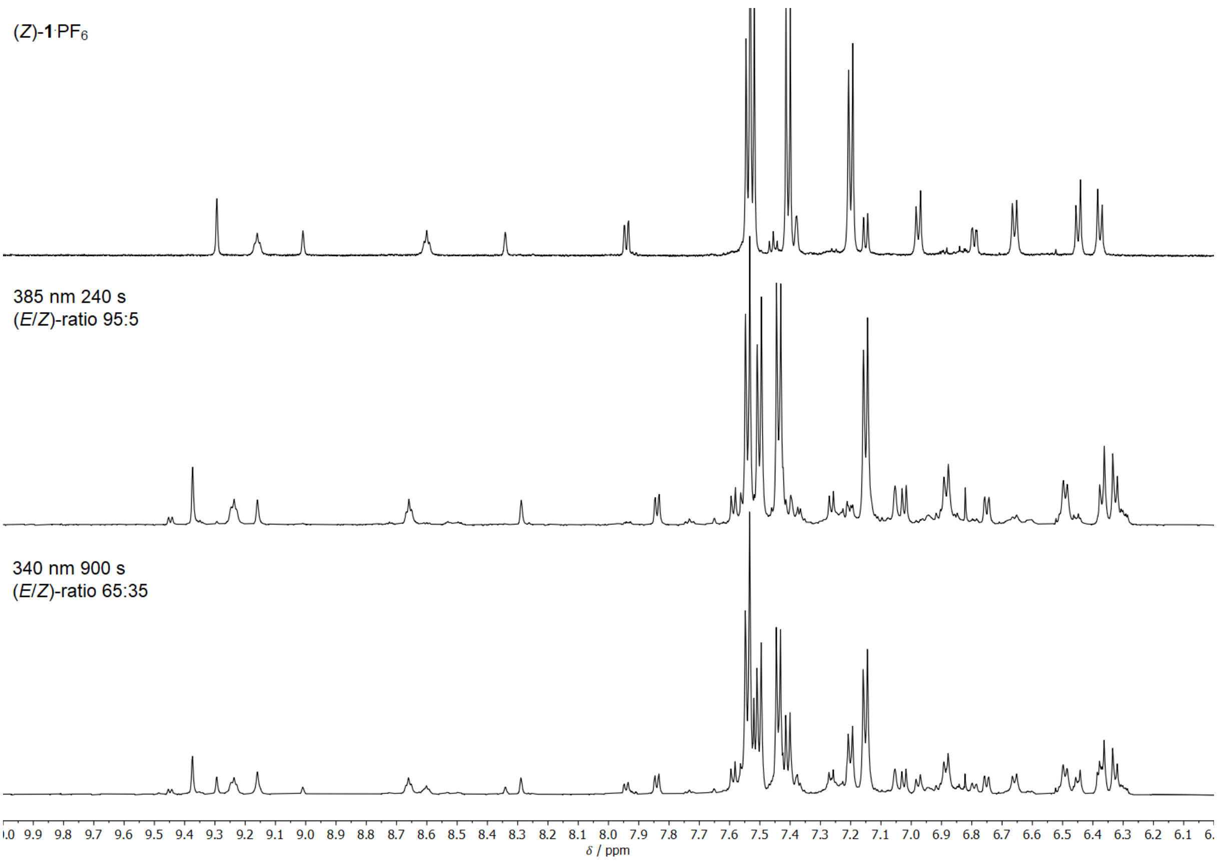

Figure S25: ^{1}H NMR spectral changes (600 MHz, 298 K) in the downfield region of (*Z*)-**1**·PF_6 (0.20 mM in DMSO-*d*$_6$) upon irradiation with 385 nm light for 240 s, followed by 340 nm light for 900 s.

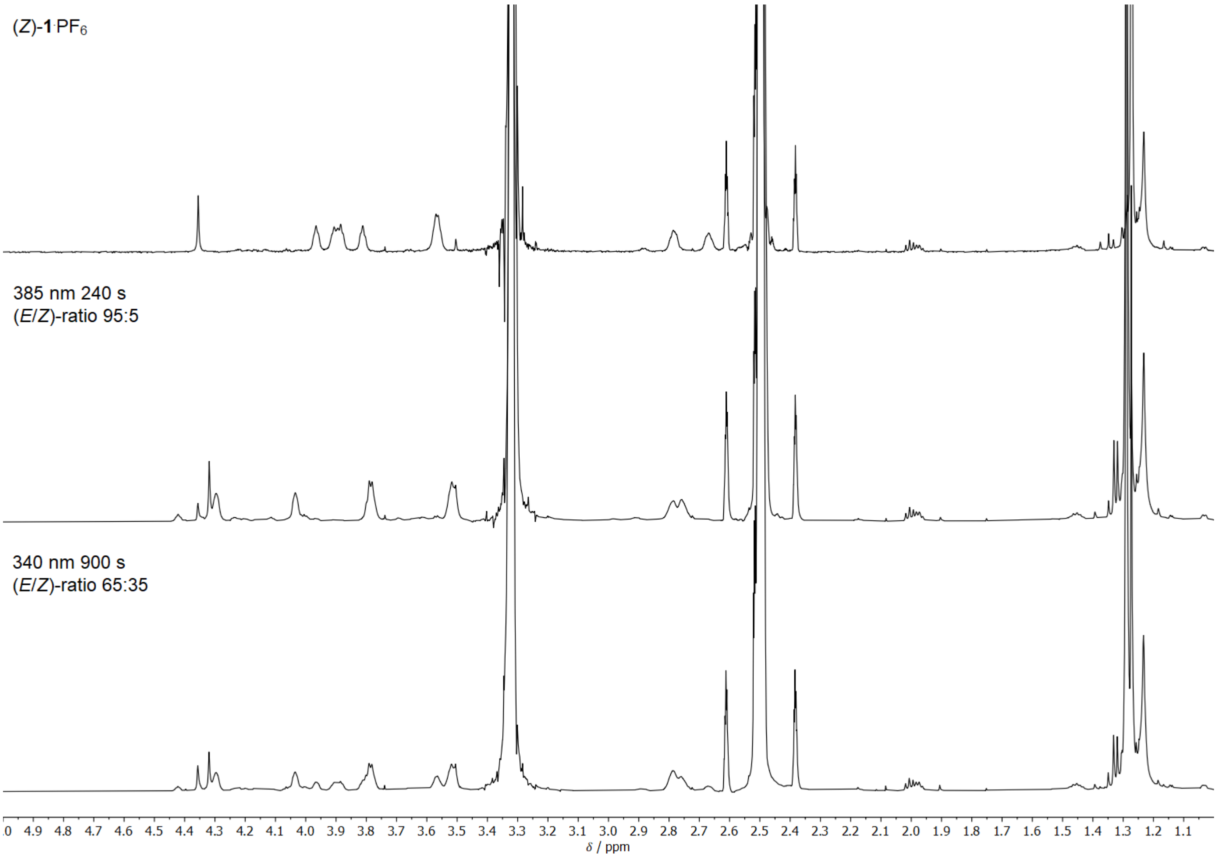

Figure S26: ^{1}H NMR spectral changes (600 MHz, 298 K) in the upfield region of (*Z*)-**1**·PF_6 (0.20 mM in DMSO-*d*$_6$) upon irradiation with 385 nm light for 240 s, followed by 340 nm light for 900 s.

Variable-temperature ^{1}H NMR experiments

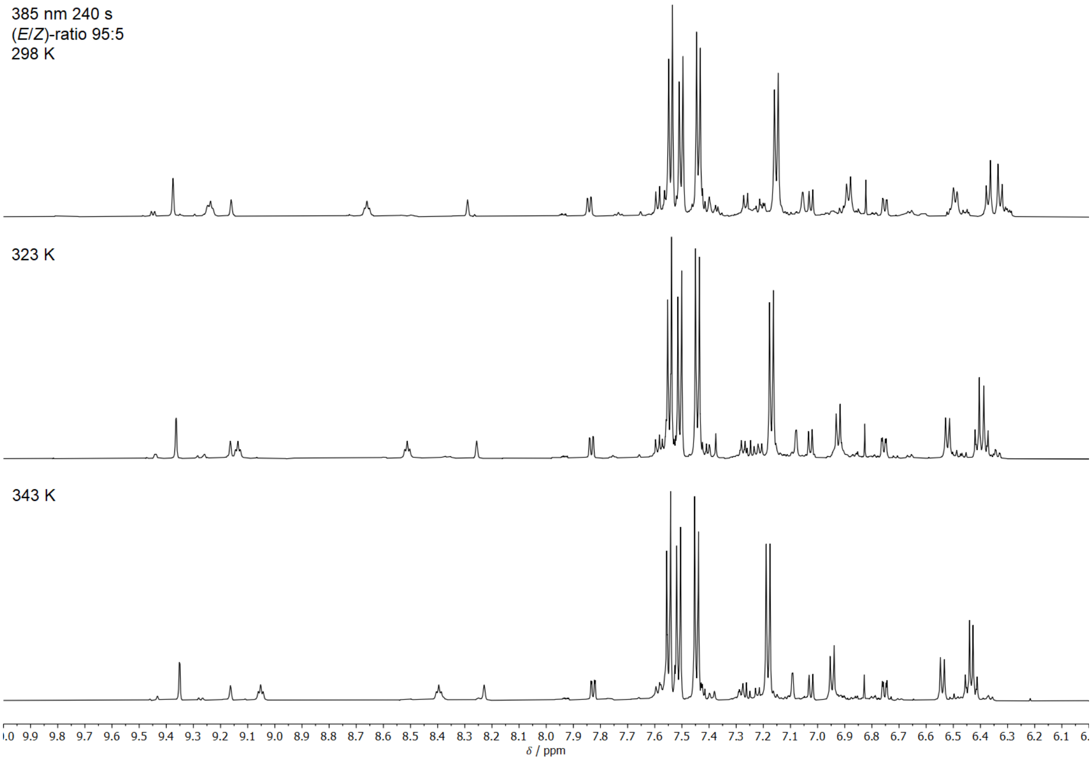

Figure S27: Selected downfield region in the ^{1}H NMR spectrum (600 MHz, 298 K) of (*E*)-**1**·PF_6 (0.2 mM in DMSO-d_6) recorded at different temperatures.

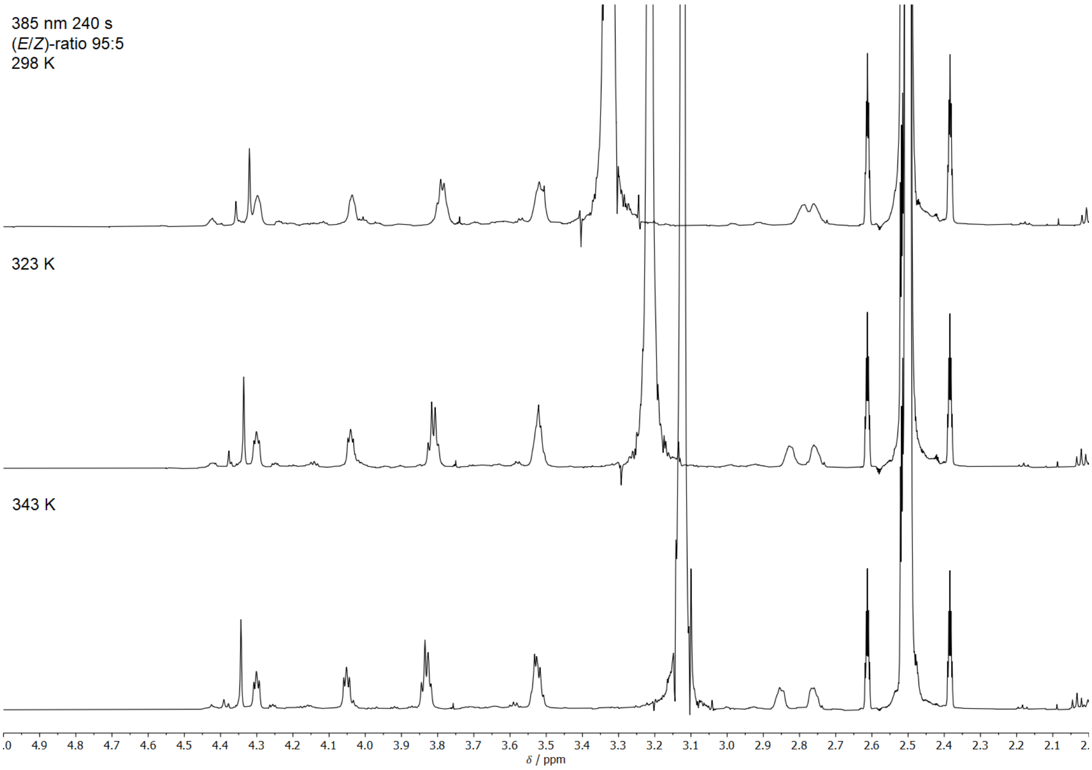

Figure S28: Selected upfield region in the ^{1}H NMR spectrum (600 MHz, 298 K) of (*E*)-**1**·PF_6 (0.2 mM in DMSO-d_6) recorded at different temperatures.

^{1}H NMR titration experiments

For the ^{1}H NMR titration experiment with (*Z*)-**1**·PF_6, a 0.50 mM solution was prepared in 1 mL DMSO-d_6 containing 0.5% (v/v) H_2O. Of this solution, 0.5 mL was used to dissolve tetrabutylammonium chloride (NBu_4Cl) and this guest solution was added stepwise to 0.5 mL of the host solution, and a ^{1}H NMR spectrum (600 MHz, 298 K) was recorded after each addition.

For the titration experiment with (*E*)-**1**·PF_6, a 0.50 solution of the *Z*-isomer was prepared in 995 µL DMSO-d_6 that was purged with nitrogen gas for 30 min. This solution was then irradiated for 10 minutes with 385 nm light, after which 5 µL of H_2O was added to get to a total volume of 1 mL. Of this solution, 0.5 mL was used to dissolve NBu_4Cl and this guest solution was added stepwise to 0.5 mL of the host solution, and a ^{1}H NMR spectrum (600 MHz, 298 K) was recorded after each addition.

The ^{1}H NMR titration data was fitted to a 1 : 1 binding model using HypNMR software.[4] Errors are estimated to be no more than ±15%.

Addition of NBu_4Cl to (*Z*)-1·PF_6 in DMSO-d_6/0.5%H_2O:

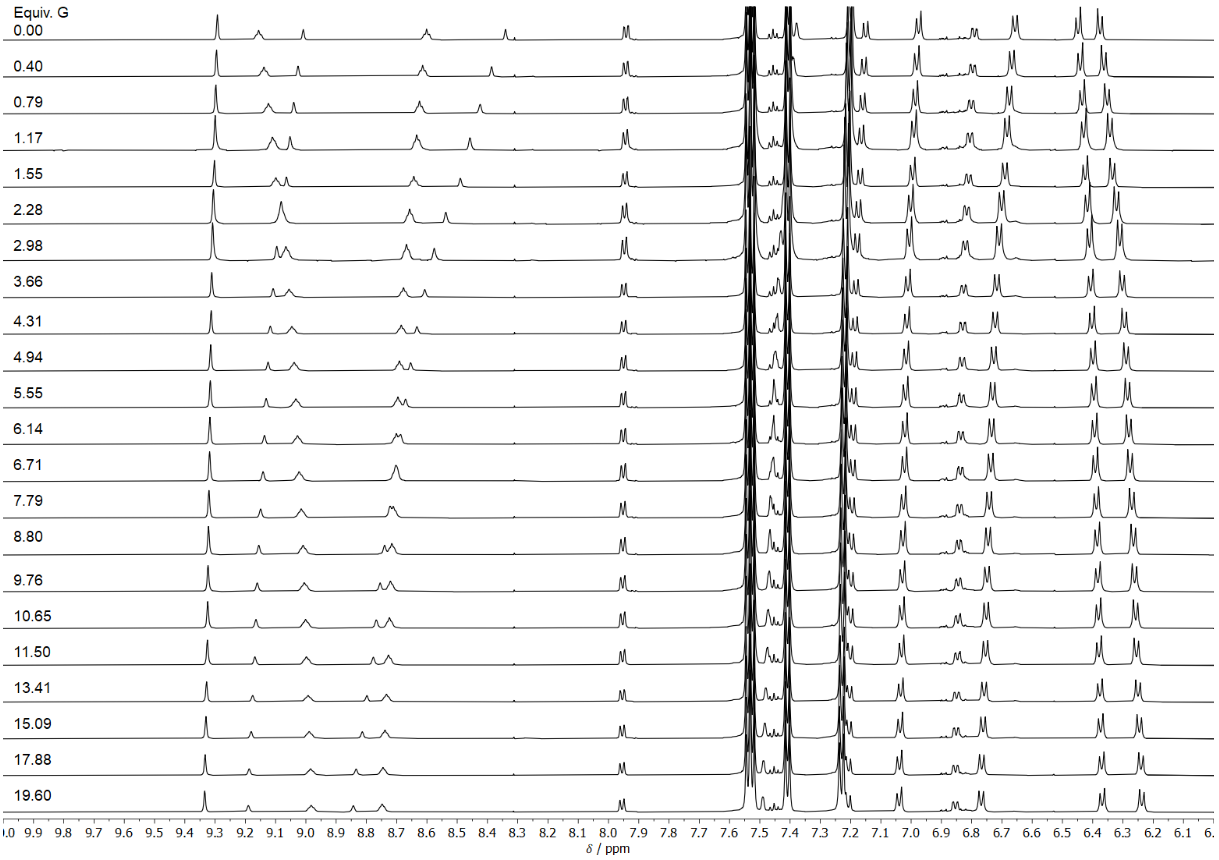

Figure S29: ^{1}H NMR spectral changes (600 MHz, 298 K) in the downfield region of (*Z*)-**1**·PF_6 (0.50 mM in DMSO-d_6/0.5%H_2O) upon the stepwise addition of a 20 mM solution of tetrabutylammonium chloride.

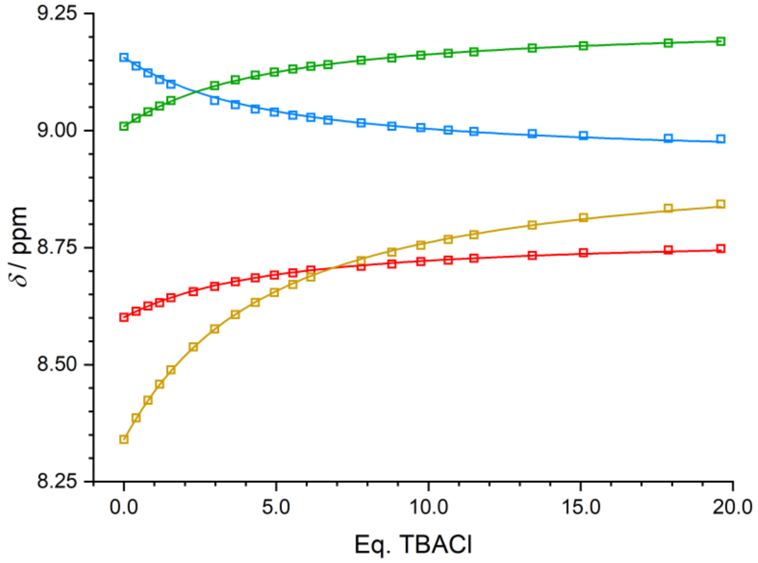

Figure S30: Chemical shift of the pyridinium NH and CH protons (blue, green), as well as the isophthalamide NH and CH protons (red, yellow) of (*Z*)-**1** during the titration and curve fitting obtained by using a 1 : 1 binding model with HypNMR software; $K_a = 4.9 \times 10^2$ M^{-1}.

Addition of NBu_4Cl to (E)-1·PF_6 in DMSO-d_6/0.5%H_2O:

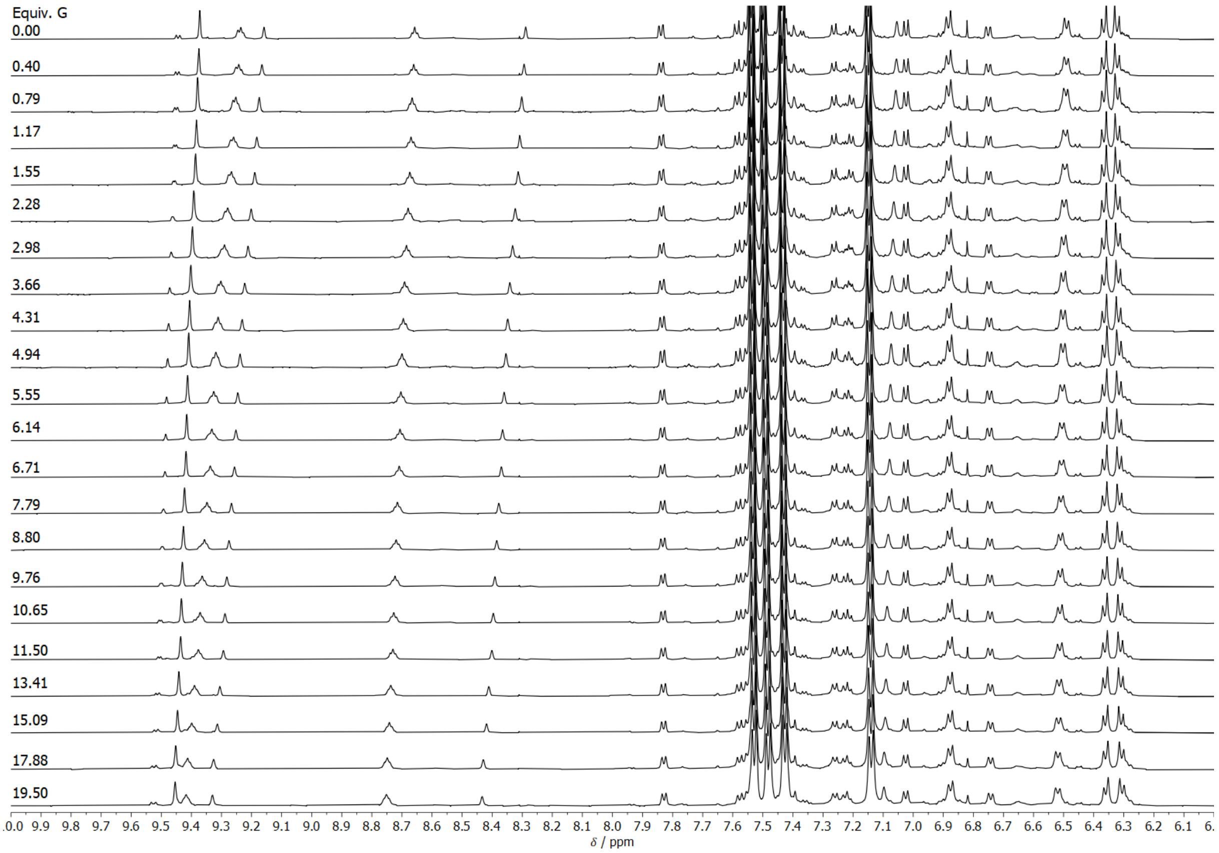

Figure S31: ^{1}H NMR spectral changes (600 MHz, 298 K) in the downfield region of (E)-**1**·PF_6 (0.50 mM in DMSO-d_6/0.5%H_2O) upon the stepwise addition of a 20 mM solution of tetrabutylammonium chloride.

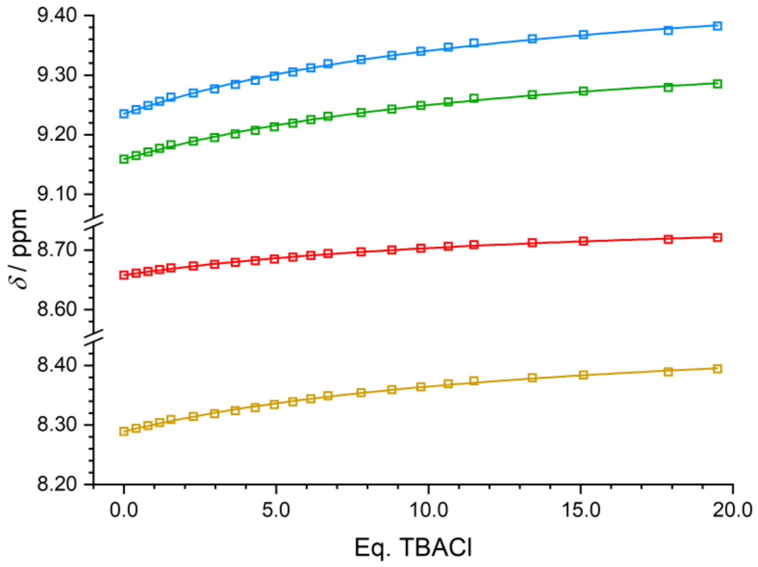

Figure S32: Chemical shift of the pyridinium NH and CH protons (blue, green), as well as the isophthalamide NH and CH protons (red, yellow) of (E)-**1** during the titration and curve fitting obtained by using a 1 : 1 binding model with HypNMR software; $K_a = 1.5 \times 10^2$ M^{-1}.

^{1}H NMR irradiation in presence of chloride

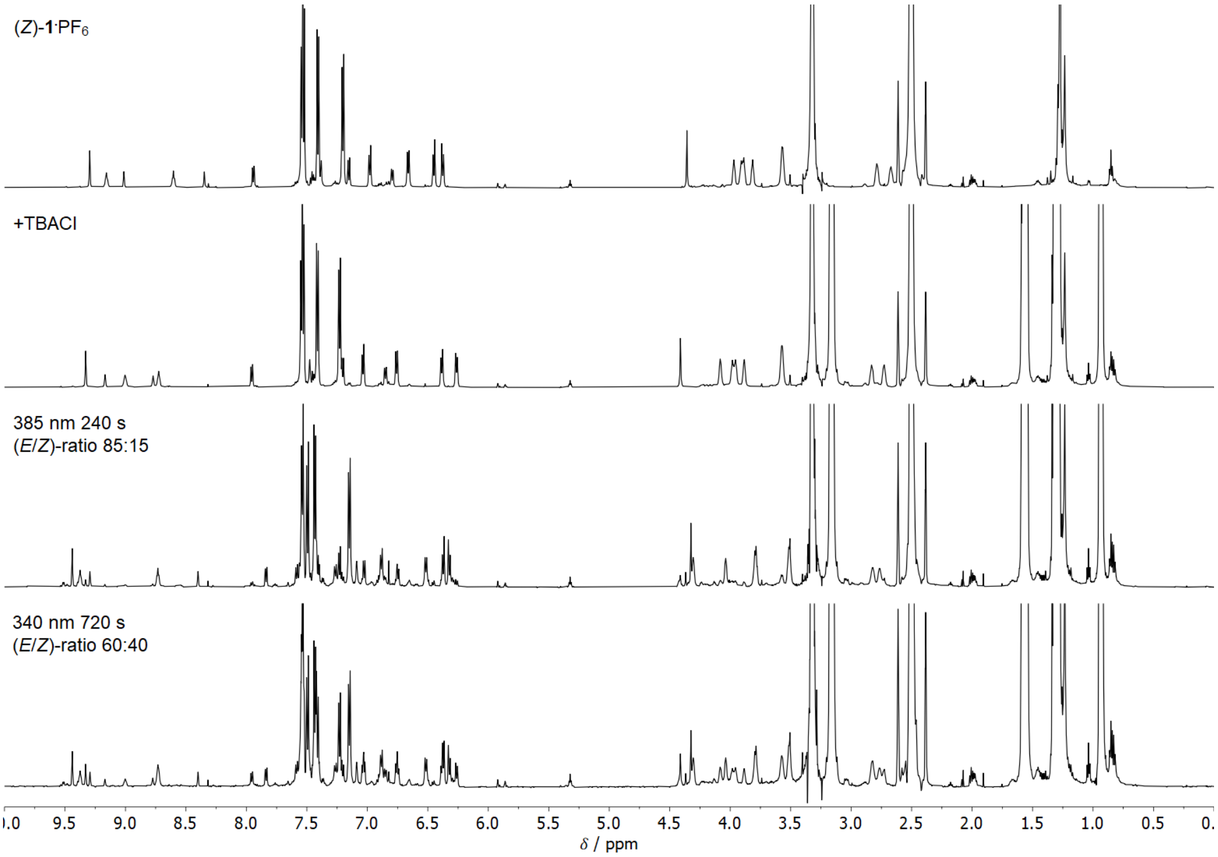

Figure S33: ^{1}H NMR spectral changes (600 MHz, 298 K) of a mixture of (*Z*)-**1**·PF_6 (0.2 mM in degassed DMSO-d_6) after addition of 20 equivalents of tetrabutylammonium chloride, followed by sequential irradiation with 385 nm light for 240 s and 340 nm light for 720 s.

References

[1] H. Kawai, T. Umehara, K. Fujiwara, T. Tsuji, T. Suzuki, *Angew. Chem. Int. Ed.* **2006**, *45*, 4281–4286.

[2] L. C. Gilday, N. G. White, P. D. Beer, *Supramol. Chem.* **2016**, *28*, 62–83.

[3] J. de Jong, M. A. Siegler, S. J. Wezenberg, *Angew. Chem. Int. Ed.* **2024**, *63*, e202316628.

[4] C. Frassineti, S. Ghelli, P. Gans, A. Sabatini, M. S. Moruzzi, A. Vacca, *Anal. Biochem.* **1995**, *231*, 374–382.
